# A Multicentre, Double-Blind, Randomised, Non-Inferiority Trial of a Novel Single-Injection Intra-Articular HMDA-Cross-Linked Hyaluronate Gel for Knee Osteoarthritis

**DOI:** 10.3390/jcm14124384

**Published:** 2025-06-19

**Authors:** Kang-Il Kim, Yong In, Hyung-Suk Choi, Ju-Hong Lee, Jae-Ang Sim, Han-Jun Lee, Young-Wan Moon, Oog-Jin Shon, Jong-Keun Seon, Young-Mo Kim, Sang-Jun Song, Chong-Bum Chang, Hyuk-Soo Han

**Affiliations:** 1 Department of Orthopaedic Surgery, Center for Joint Diseases and Rheumatism, Kyung Hee University Hospital at Gangdong, 892 Dongnam-ro, Gang-dong-gu, Seoul 05278, Republic of Korea; 2 Department of Orthopaedic Surgery, Seoul St. Mary’s Hospital, College of Medicine, The Catholic University of Korea, Seoul 06591, Republic of Korea; iy1000@catholic.ac.kr; 3 Department of Orthopaedic Surgery, Soonchunhyang University Seoul Hospital, Seoul 04401, Republic of Korea; knee@schmc.ac.kr; 4 Department of Orthopaedic Surgery, Chonbuk National University Hospital, Jeonju 54907, Republic of Korea; jhlee55@jbnu.ac.kr; 5 Department of Orthopaedic Surgery, Gachon University Gil Hospital, Gil Medical Center, Incheon 21565, Republic of Korea; sim_ja@daum.net; 6 Department of Orthopaedic Surgery, Chung-Ang University College of Medicine, Seoul 06973, Republic of Korea; gustinolhj@nate.com; 7 Department of Orthopaedic Surgery, Samsung Medical Center, Sungkyunkwan University School of Medicine, Seoul 06351, Republic of Korea; yw1.moon@samsung.com; 8 Department of Orthopaedic Surgery, Yeungnam University College of Medicine, Daegu 42415, Republic of Korea; ossoj@med.yu.ac.kr; 9 Department of Orthopaedic Surgery, Chonnam National University Hwasun Hospital, Hwasun-gun 58128, Republic of Korea; seonbell@jnu.ac.kr; 10 Department of Orthopaedic Surgery, Chungnam National University Hospital, Daejeon 35015, Republic of Korea; osdr69@gmail.com; 11 Department of Orthopaedic Surgery, Kyung Hee University Medical Center, Seoul 02447, Republic of Korea; tesstore@empas.com; 12 Department of Orthopaedic Surgery, Seoul National University Bundang Hospital, Seongnam-si 13620, Republic of Korea; ccbknee@gmail.com; 13 Department of Orthopaedic Surgery/Stem Cell Biology, Seoul National University College of Medicine, Seoul 03080, Republic of Korea

**Keywords:** knee osteoarthritis, intra-articular injection, treatment, hyaluronic acid, hexamethylenediamine

## Abstract

**Background/Objectives**: This Phase 3, randomised, double-blind, multicentre trial evaluated the efficacy and safety of a novel hyaluronic acid hydrogel cross-linked with hexamethylenediamine (HMDA-HA) compared to a conventional 1,4-butanediol diglycidyl ether cross-linked HA (BDDE-HA) in patients with mild-to-moderate knee osteoarthritis (OA). **Methods**: A total of 223 adults (mean age 63.5 years; 167 women) with Kellgren–Lawrence (KL) grade I–III knee OA were randomised 1:1 to receive two intra-articular injections of HMDA-HA or BDDE-HA at baseline and at 24 weeks. The primary endpoint was changes from baseline in weight-bearing pain (WBP) on a 100 mm visual analogue scale (VAS) at Week 12, assessed in the per-protocol population. A non-inferiority margin of 10 mm was predefined. Secondary outcomes included global assessments, Western Ontario and McMaster Universities Osteoarthritis (WOMAC) index scores, responder rates, and rescue medication use [ClinicalTrials.gov: NCT06307847]. **Results**: At Week 12, least squares mean change (standard error [SE]) in WBP was −23.72 (1.88) mm in the HMDA-HA group (n = 83) and −25.99 (1.76) mm in the BDDE-HA group (n = 95), yielding a difference of 2.26 mm (95% confidence interval [CI]: −2.83 to 7.34; *p* = 0.3825), thus demonstrating the non-inferiority of HMDA-HA to BDDE-HA. Secondary outcomes were comparable between groups. A total of 136 adverse events were reported: 44 (41.1%) in the HMDA-HA group and 32 (28.1%) in the BDDE-HA group, with no treatment-related adverse drug reactions. **Conclusions**: A single-injection intra-articular regimen of HMDA-HA was effective and safe for the treatment of adult patients with mild-to-moderate knee OA.

## 1. Introduction

Osteoarthritis (OA) is a progressive disease affecting synovial joints, with a lifetime risk over 40% and representing a leading cause of disability and socioeconomic burden in older adults [1]. OA is characterized by a multifactorial degeneration of articular cartilage, accompanied by concomitant changes in synovial tissue and subchondral bone metabolism [1]. Intra-articular hyaluronic acid (IAHA) injections are recommended for the treatment of OA due to their favourable safety profile, particularly for older patients with knee OA or those at increased risk for adverse events (AEs) associated with nonsteroidal anti-inflammatory drugs (NSAIDs). Recent guidelines from the Osteoarthritis Research Society International (OARSI) and the European Society for Clinical and Economic Aspects of Osteoporosis, Osteoarthritis, and Musculoskeletal Diseases (ESCEO) advocate the use of IAHA in patients with knee OA with contraindications to NSAIDs or following NSAID treatment failure [2,3] The viscosupplementation of knee OA with IAHA has demonstrated clinical benefits in pain relief, functional improvement, and patient-reported outcomes [4,5,6,7].

Hyaluronic acid (HA) is a naturally occurring glycosaminoglycan composed of repeating units of β-1,3-*N*-acetylglucosamine and β-1,4-D-glucuronic acid [8]. HA is a key component of synovial fluid, contributing to joint lubrication, viscoelastic shock absorption, and proteoglycan synthesis, while also exerting chondroprotective, anti-inflammatory, and analgesic effects [9,10,11]. In patients with knee OA, synovial fluid exhibits decreased HA concentration and increased levels of hyaluronidases, inflammatory cytokines, and free radicals including reactive oxygen species, leading to further impaired HA function and contributing to disease progression [8]. However, unmodified, natural linear HA is rapidly degraded by endogenous hyaluronidases and eliminated from the joint within a few days [8]. IAHA formulations using unmodified HA have therefore required frequent injection schedules, which may cause synovial membrane damage and inconvenience to patients due to repeated medical visits. To overcome the limitation of low biological persistence, cross-linked HA hydrogels have been introduced for IAHA injections, employing cross-linking agents such as 1,4-butanedioldiglycidylether (BDDE) and divinyl sulfone (DVS) [12]. Although effective, these crosslinkers react with hydroxyl groups of HA, leaving the carboxyl groups—primary targets of hyaluronidase—unprotected [13].

**Figure 1:**
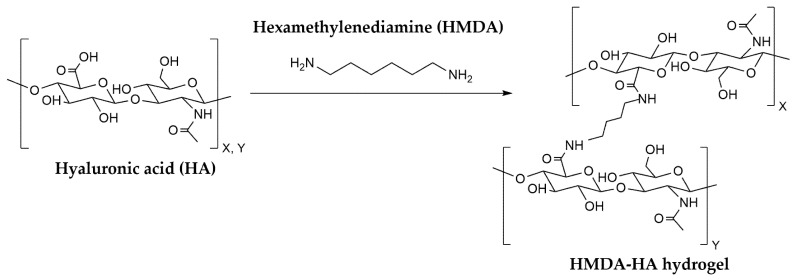
Synthetic scheme of HMDA-HA hydrogel.

This study introduces a novel, autoclavable cross-linked HA hydrogel [14], using a new and potentially safer cross-linking agent, 1,6-hexamethylenediamine (HMDA; also known as 1,6-diaminohexane or 1,6-hexadiamine) [15]. By forming stable amide bonds with the carboxyl groups of HA (Figure 1), HMDA-mediated cross-linking enhances its resistance to enzymatic degradation [13,16]. The resulting HMDA-cross-linked HA (HMDA-HA) hydrogel demonstrates superior stability against hyaluronidase-mediated breakdown compared to BDDE- or DVS-cross-linked HA [16,17,18,19]. In a rat pharmacokinetic study using a ^14^C-labeled HMDA-HA hydrogel, 67.5% of the injected hydrogel remained at the injection site after 70 days [17], consistent with the characteristics of a locally acting formulation. The estimated intra-articular half-life was approximately 123.5 days in rats [17]—substantially longer than the <2-day half-life of unmodified HA [11,17,19,20]. HMDA degrades into non-toxic metabolites such as adipic acid or fatty acids, which enter the tricarboxylic acid (TCA) cycle as energy substrates [15]. Human metabolic studies have shown that the administered HMDA is rapidly excreted via the kidneys, either unchanged or as 6-aminohexanoic acid [21,22,23]. HMDA is not expected to generate persistent toxic residues, making it a potentially safer alternative—even in the formation of reactive pendant cross-linking structures, as reported in hydrogels cross-linked with BDDE and DVS [24,25]. Furthermore, HMDA-HA exhibits enhanced elasticity (storage modulus (723 vs. 246 Pa for BDDE-HA; approximately 2.9-fold) and shear viscosity (484 vs. 286 Pa·s; ~1.7-fold) (unpublished in vitro data), as well as improved thermal stability, retaining its physicochemical properties after autoclave sterilisation [26].

The nonclinical efficacy of HMDA-HA was demonstrated in a rabbit model of surgically induced knee OA involving anterior cruciate ligament transection and partial medial meniscectomy [17]. Intra-articular HMDA-HA alleviated OA-associated pathological changes, including joint swelling and capsule thickening, and preserved knee extension. Histopathological analysis showed maintained cartilage thickness and chondrocyte density in femoral and tibial regions, and reduced Mankin scores. HMDA-HA also significantly reduced synovial IL-1β and TNF-α levels and decreased IL-1β- and TNF-α-immunopositive cells in cartilage. Comprehensive toxicological assessment included single- and repeat-dose toxicity studies of up to 28 weeks in rats and 40 weeks in dogs; genotoxicity studies using both bacterial and mammalian systems; a 26-week repeat-dose carcinogenicity study of HMDA-HA and HMDA in CB6F1-Tg rasH2 transgenic mice; and antigenicity studies in guinea pigs [17]. Reproductive toxicity was previously reported in rats [15,27].

A multicentre, randomised, double-blind, placebo-controlled Phase 1/2 trial (SP-HA-001) was conducted in South Korea to evaluate the safety and efficacy of HMDA-HA [17]. The investigational intra-articular HMDA-HA formulation was designed to mimic the viscoelastic properties of healthy human synovial fluid (elasticity/storage modulus: 117 ± 13 Pa at 2.5 Hz for individuals aged 21–45 years) [28], accounting for at least two-fold dilution upon intra-articular injection into older adults (aged 52–78 years; 19 ± 3 Pa), younger OA patients (aged 21–45 years, 1.9 Pa at 2.5 Hz), or patients with moderate OA who had undergone total knee arthroplasty (1.9 ± 0.5 Pa at 2.5 Hz) [28,29,30,31], and considering an average synovial fluid volume of 6.7 ± 2.3 mL [32]. Patients with knee OA were randomised to receive 3 mL or 5 mL of HMDA-HA, or placebo. Although the primary endpoint—change in weight-bearing pain (WBP) on a 100 mm visual analogue scale (VAS)—was not met due to the limited sample size, post-hoc analysis revealed a significant reduction in pain at Week 24 in the 5 mL HMDA-HA group, which was subsequently selected as the clinical dose for the Phase 3 study. Improvements were also observed in Western Ontario and McMaster Universities Osteoarthritis (WOMAC) index scores, and no notable safety concerns were reported.

Building upon these findings, this multicentre, randomised, double-blind, active-controlled Phase 3 study was designed to evaluate the clinical efficacy and safety of a single-injection intra-articular HMDA-HA regimen administered at 6-month intervals in patients with mild-to-moderate knee OA. Although a longer follow-up may be scientifically desirable given the expected prolonged intra-articular residence of HMDA-HA, the trial was conducted under a conservative design, as required by regulatory authorities for a first-in-human study. Accordingly, the study adopted a parallel-group comparison with commercially available BDDE-HA, allowing a second injection at Week 24 per its approved dosing schedule, followed by a 12-week observation period through Week 36. The primary objective was to determine whether HMDA-HA is non-inferior to BDDE-crosslinked HA in reducing pain, as measured by change from baseline in WBP at Week 12, with a predefined non-inferiority margin of 10 mm [33]. Secondary endpoints included additional pain domains, functional outcomes, patient and investigator global assessments, and safety profiles.

## 2. Materials and Methods

### 2.1. Study Design and Participants

Eligible participants were adults aged ≥40 years who met the American College of Rheumatology clinical criteria for mild-to-moderate knee osteoarthritis (OA) and had radiographic evidence of Grade I to III OA according to the Kellgren and Lawrence (KL) grading scale [13]. Individuals with a body mass index (BMI) ≥35 kg/m^2^, OA involving joints other than the knee, or any comorbid conditions—including active infections or dermatologic diseases—that could interfere with efficacy or safety assessments were excluded. Additional exclusion criteria included systemic steroid use within the prior 3 months or intra-articular corticosteroid or HA injection within 6 months prior to screening. Detailed inclusion and exclusion criteria are provided in Table S1.

In participants with unilateral knee OA, the affected knee was designated as the target study knee. For participants with bilateral OA, the target knee was defined as the one with greater WBP on the VAS at screening. If both knees had identical WBP scores, the right knee was selected.

### 2.2. Trial Oversight

The study protocol was reviewed and approved by independent institutional review boards (IRBs) at each study site. All participants provided written informed consent before undergoing any study-related procedures. The study was conducted in accordance with the International Council for Harmonisation of technical requirements for pharmaceuticals for human use (ICH) Good Clinical Practice guidelines, the principles of the Declaration of Helsinki, and all applicable local regulatory requirements. This trial was registered on ClinicalTrials.gov (NCT06307847) on 13 March 2024 (https://clinicaltrials.gov/study/NCT06307847; accessed on 16 June 2025).

### 2.3. Randomisation and Masking

Screening occurred up to 4 weeks before randomisation and included a mandatory 2-week washout period for participants who had received prohibited medications. Following informed consent and screening, eligible participants were randomly assigned in a 1:1 ratio to receive a single intra-articular injection of either HMDA-HA or BDDE-HA. Randomisation was stratified by site and implemented using a block randomisation method generated via SAS version 9.4 (SAS Institute Inc., Cary, NC, USA). Participants, investigators, monitors, and study personnel remained blinded throughout the study. To maintain masking, injections were administered by independent unblinded investigators, while all efficacy assessments were conducted by blinded assessors.

### 2.4. Study Intervention

The HMDA-HA hydrogel (code: SP5M001; brand name: Hyalflex^®^; 5 mL per prefilled syringe, containing 100 mg of sodium hyaluronate cross-linked with HMDA) was manufactured using non-animal, biological fermentation-derived HA under Good Manufacturing Practice (GMP) conditions by Shin Poong Pharm. Co., Ltd. (Ansan, Republic of Korea) [17] (Supplementary Materials S3). The active comparator, BDDE-HA (Synovian^®^; 3 mL gel per prefilled syringe), was manufactured by LG Chem, Ltd. (Seoul, Republic of Korea).

Following a 2-week washout period after screening, participants received a single intra-articular injection of either HMDA-HA or BDDE-HA into the target knee at baseline (Day 1, Week 0). Injections were administered under aseptic conditions using 21-gauge needles. A second single intra-articular injection of the same study intervention was administered at Week 24.

Acetaminophen (≤4 g/day) was permitted as rescue analgesia but was prohibited within 24 h prior to each pain assessment. Low-dose aspirin (≤300 mg/day) for cardiovascular prevention was also allowed, except within 24 h prior to assessments. Concomitant medications, such as antihypertensive or antidiabetic agents, were permitted at the discretion of the investigator if deemed not to interfere with study outcomes. All other treatments potentially affecting the study results—including NSAIDs, steroids, analgesics, anaesthetics, intra-articular injections, and OA-specific supplements—were prohibited throughout the study period.

### 2.5. Assessments and Outcomes

Follow-up visits for efficacy and safety assessments were conducted at Weeks 2, 6, 12, 24, and 36. The end-of-study (EOS) visit was conducted at Week 36, 12 weeks after the second injection. In cases of early termination, withdrawal, or discontinuation, all assessments scheduled for the end-of-study visit were conducted at the time of discontinuation

The primary efficacy endpoint was the change in WBP measured on a 100 mm VAS from baseline to Week 12 following the initial treatment. Secondary efficacy outcomes, assessed at Weeks 2, 6, 12, 24, and 36, included the following: changes from baseline in WBP, rest pain, night pain, motion pain, patient global assessment (PGA), and investigator global assessment (IGA), each measured by a 100 mm VAS; the WOMAC total score and its subscales (pain, stiffness, and function) [34]; the range of motion, swelling, and tenderness of the target knee; the proportion of patients using rescue medication and the total consumption of rescue medicines; WBP responder rate, defined as the proportion of participants achieving at least a 20 mm or 40% reduction in WBP from baseline; and the OMERACT-OARSI responder rate [35]. OMERACT-OARSI responders were defined as participants meeting one of the following criteria: (1) ≥50% improvement or a ≥20-point improvement in either WOMAC pain or function score; or (2) improvement in at least two of the following three: (i) ≥20% or ≥10-point improvement in WOMAC pain score; (ii) ≥20% or ≥10-point improvement in WOMAC function score; and (iii) ≥20% or ≥10-point improvement in PGA (measured by 100 mm VAS).

Safety was assessed by collecting adverse events (AEs) at each visit based on investigator observations, participant self-reports, vital signs, and laboratory findings. All AEs, including adverse drug reactions (ADRs), were recorded regardless of severity or causality and classified using the Medical Dictionary for Regulatory Activities (MedDRA), version 25.1. Solicited local AEs at the injection site (e.g., erythema, swelling, pain, and warmth) were recorded using patient diaries for 7 days after each injection.

### 2.6. Statistical Analysis

Safety analyses were performed using the safety set, comprising randomised participants who received at least one dose of the study intervention. Efficacy analyses were conducted using both the full analysis set (FAS) and the per-protocol set (PPS), with the PPS serving as the primary analysis set. The FAS comprised participants who received at least one dose and had the primary efficacy assessment, whereas the PPS excluded participants with major protocol deviations, as determined by a case review prior to unblinding.

The primary efficacy objective was to demonstrate the non-inferiority of HMDA-HA compared with BDDE-HA. The primary endpoint—change from baseline in WBP at Week 12—was analysed using an analysis of covariance (ANCOVA), with the treatment group as a fixed effect and baseline WBP as a covariate. Least squares means (LS means; HMDA-HA minus BDDE-HA) and two-sided 95% confidence intervals (CIs) for between-group differences were estimated. Based on previous BDDE-HA trial data [33], the standard deviation (SD) of WBP change was assumed to be 22 mm. A sample size of 77 participants per group (1:1 allocation) was calculated to provide 80% power to demonstrate non-inferiority (non-inferiority margin: 10 mm; one-sided alpha = 0.025). Allowing for a 30% dropout rate and protocol violations, the final sample size was set at 220 (110 per group; refer to Supplementary Materials S1).

Secondary pain endpoints assessed by a 100 mm VAS were analysed using the same ANCOVA model. Other continuous secondary outcomes were analysed using a two-sample *t*-test or the Wilcoxon rank-sum test, as appropriate. Categorical variables were analysed using chi-square or Fisher’s exact test. For efficacy analyses, missing data in the FAS due to early withdrawal or other reasons were imputed using the last observation carried forward (LOCF) method. All analyses were performed using SAS^®^ version 9.4 (SAS Institute Inc., Cary, NC, USA).

## 3. Results

### 3.1. Patient Disposition

**Figure 2:**
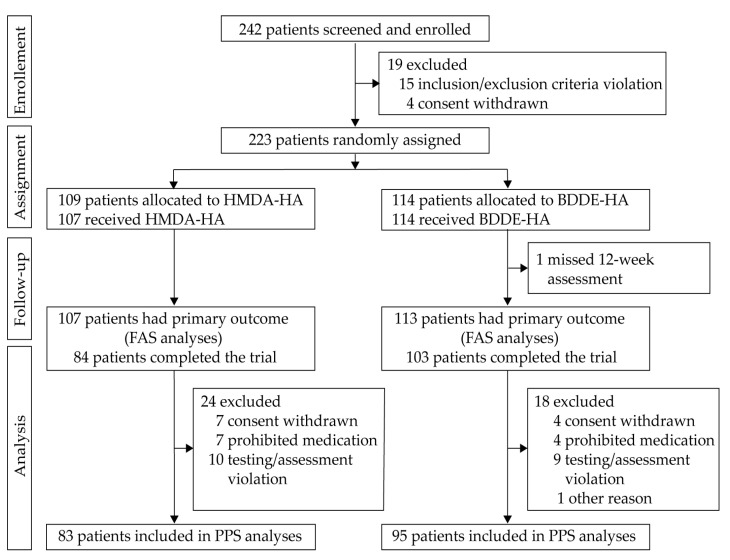
Participant flow.

Between 12 April 2021 and 25 October 2022, 242 participants were screened at 14 sites in South Korea. Of these, 223 were randomised to receive the study intervention, with 109 assigned to the HMDA-HA group and 114 to the BDDE-HA group (Figure 2). A total of 221 participants received at least one dose of the study intervention, with 193 receiving the scheduled second dose at Week 24. The full analysis set (FAS) included 220 participants, and 187 completed the 36-week follow-up. The per-protocol set (PPS), used for primary efficacy analysis, included 178 participants (83 HMDA-HA; 95 BDDE-HA) without major protocol deviations.

Demographic and baseline characteristics were well balanced between groups (Table 1 and Table S2). The mean age of participants was 63.48 years, with a predominance of women (75.91%). KL grade II was most prevalent (53.2%), followed by grade III (34.6%) and grade I (12.3%).

### 3.2. Efficacy Outcomes

#### 3.2.1. Primary Efficacy Endpoint

**Figure 3:**
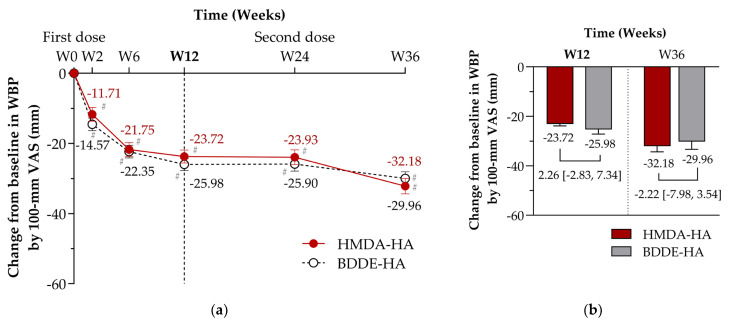
Changes from baseline in weight-bearing pain (WBP) on a 100 mm visual analogue scale (VAS) through Week 36 (per-protocol set). Participants received intra-articular injections at baseline (Week 0) and again at Week 24. The graph and error bars represent least squares mean (LS mean) changes and standard errors (SEs) in WBP for the HMDA-HA group (red solid line) and the BDDE-HA group (black dashed line). Data labels indicate LS mean change in millimetres (mm) at each time point. (**a**) Time-course of changes from baseline in WBP at Weeks 2, 6, 12, 24, and 36. The primary efficacy endpoint was assessed at Week 12, with the predefined non-inferiority analysis indicated by the vertical dashed line. Within-group comparisons using paired *t*-tests showed statistically significant reductions in WBP from baseline at all time points for both groups (^#^ *p* < 0.0001), indicating sustained improvement. (**b**) LS mean changes from baseline in WBP at Weeks 12 and 36, with between-group differences and corresponding 95% confidence intervals (CI). At Week 12, the LS mean difference (HMDA-HA minus BDDE-HA) was 2.26 mm [95% CI: −2.83 to 7.34], meeting the criterion for non-inferiority based on the pre-specified margin of 10 mm. At Week 36 (12 weeks after the second dose), the difference was −2.22 mm [95% CI: −7.98 to 3.54], suggesting a potential trend toward greater pain improvements in the HMDA-HA group following re-dosing.

The primary endpoint was changes from baseline in WBP on a 100 mm VAS at Week 12, assessed in the PPS. Both the HMDA-HA group and the BDDE-HA group showed progressive reductions in WBP over time (Figure 3). At Week 12, the adjusted LS mean changes from baseline in WBP showed a significant reduction from baseline as −23.72 mm (standard error [SE], 1.88; *p* <0.0001) in the HMDA-HA group and −25.98 mm (1.76; *p* < 0.0001) in the BDDE-HA group (Figure 3, Table 2). The between-group LS mean difference (HMDA-HA minus BDDE-HA) was 2.26 mm [95% CI: −2.83, 7.34], meeting the criterion for non-inferiority based on the pre-specified margin of 10 mm. These findings were consistent across subgroups stratified by age, sex, and KL grade in post-hoc analyses (Table S3), confirming the non-inferiority of HMDA-HA to BDDE-HA.

#### 3.2.2. Secondary Efficacy Endpoints

Both treatment groups—HMDA-HA and BDDE-HA—demonstrated statistically significant improvements across multiple efficacy endpoints over the 36-week study period, with no significant differences observed between groups at any visit (Figure 3, Table 2, Table S4 and Table S5). These findings were consistently observed in the FAS, supporting the robustness of the finding.

Significant reductions in WBP were observed at all post-baseline visits (Weeks 2–36) in both groups, with improvements further enhanced following the second injection at Week 24 (Figure 3, Table 2). At Week 36, the LS mean WBP reduction from baseline was −32.18 mm in the HMDA-HA group and −29.96 mm in the BDDE-HA group, yielding a between-group difference of −2.22 mm [95% CI: −7.98 to 3.54]. When referenced from Week 24, the difference was greater (−4.75 mm [−10.83 to 1.14]) (Table S6), favouring HMDA-HA, suggesting a potential trend toward enhanced efficacy with repeated dosing.

Other pain domains, including rest pain, night pain, and motion pain, as well as in patient and investigator global assessments measured by a 100 mm VAS, all showed statistically significant within-group improvements (*p* < 0.0001), without significant between-group differences (Table 2 and Table S4). A similar pattern in pain and functional outcomes assessed by total WOMAC scores and subscale scores for pain, stiffness, and function also supported steady improvements over time in both groups with an increased magnitude of change through Week 36.

Physical examination assessments showed improvement in joint-line tenderness and swelling in both groups (Table 2 and Table S4). A statistically significant advantage favouring HMDA-HA was observed in joint-line tenderness at Week 12 (*p* = 0.0470), but the between-group difference was not consistently maintained across other visits. Although changes were minimal and did not reach statistical significance within or between groups across any visit (Table 2 and Table S4), a numerically greater improvement in the range of motion (both extension and flexion) was observed in the HMDA-HA group through Week 36 (Figure S1, Table S4).

The consumed dose of rescue medication and the proportion of participants using rescue medication were comparable between groups (Table 2 and Table S4). The total consumed dose of rescue medication was 28.30 g in the HMDA-HA group and 31.31 g in the BDDE-HA group, with a non-significant reduction of 9.61% in the HMDA-HA group (Figure S2).

**Figure 4:**
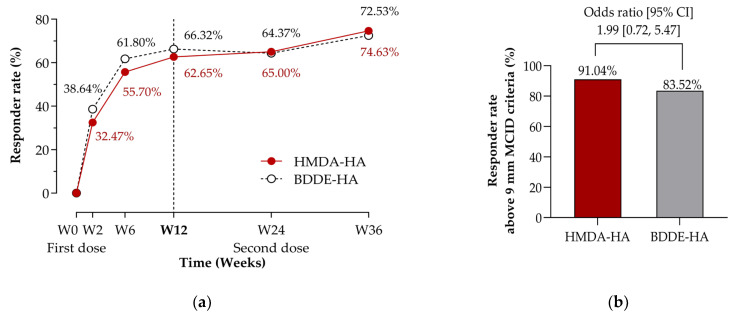
Responder rate through Week 36 (per-protocol set). Participants received intra-articular injections at baseline (Week 0) and again at Week 24. Responder rate was defined as the proportion of participants with improvement in weight-bearing pain (WBP) (%) [with a decrease in WBP measured by 100 mm VAS of ≥20 mm or improved by ≥40% from baseline]. Data labels indicate the responder rate in percentage at each time point. (**a**) The graph and error bars represent the responder rate for the HMDA-HA group (red solid line) and the BDDE-HA group (black dashed line). The vertical dashed line indicates the primary endpoint time point (Week 12). No significant differences were observed between groups at any visit by the logistic regression model. (**b**) Responder rate above the minimal clinically important difference (MCID) criteria of 9 mm in WBP at Week 36, with a between-group difference in odds ratio and corresponding 95% confidence intervals (CI).

Response rates based on predefined criteria—either a ≥20 mm or ≥40% reduction in WBP from baseline (Figure 4a, Table S5), and the OMERACT-OARSI composite responder definition (Table S5)—consistently increased over time in both groups, with no statistically significant inter-group differences at any time point. By Week 36, more than 70% of participants in both groups met the response criteria, reflecting substantial clinical benefit.

In a post-hoc analysis using an alternative minimum clinically important difference (MCID) criteria of 9 mm in WBP [36], both the HMDA-HA and BDDE-HA groups exhibited increasing responder rates over time (Table S7). Interestingly, the responder rate above MCID at Week 36 was numerically higher in the HMDA-HA group (91.04%) than in the BDDE-HA group (83.52%), corresponding to an odds ratio of 1.99 [95% CI: 0.72 to 5.47] (*p* = 0.1832) (Figure 4b; Table S7). The BDDE-HA group showed a plateau in responder rates beyond Week 24 (60.23%, 76.40%, 80.00%, 83.91%, and 83.52% at Weeks 2, 6, 12, 24, and 36, respectively), whereas the HMDA-HA group demonstrated a continued upward trend (50.65%, 77.22%, 80.72%, 78.75%, and 91.04%, respectively) (Table S7). This pattern again suggests a potentially more prolonged treatment effect of HMDA-HA following repeated administration.

### 3.3. Safety Outcomes

In the safety population (n = 221), no deaths, ADRs, or serious ADRs (SADRs) were reported during the study. Overall, 34.39% of participants experienced at least one AE, with a statistically significantly higher incidence in the HMDA-HA group (41.12%), compared to the BDDE-HA group (28.07%) (*p* = 0.0412) (Table 3). Most AEs were mild or moderate in severity and self-limiting. Four serious AEs (SAEs) occurred in the HMDA-HA group (pulmonary arteriovenous fistula, bladder neoplasm, transient ischaemic attack, and hyperkeratosis) and three in the BDDE-HA group (foot fracture, meniscus injury, and pneumonia); all events resolved and were deemed unrelated to the study treatment (Table S8).

The most frequently reported AEs fell under musculoskeletal and connective tissue disorders, followed by infections and infestations (Table 4). As the study was conducted during the COVID-19 pandemic, COVID-19 infection was the most frequently reported AE (9.35% in the HMDA-HA group vs. 5.26% in the BDDE-HA group), followed by arthralgia (10.28% vs. 1.75%), with both AEs occurring more frequently in the HMDA-HA group. A post-hoc analysis excluding COVID-19 showed no statistically significant difference in overall AE incidence between groups (35.51% vs. 25.44%; *p* = 0.1034; Table S9). Among participants with arthralgia, knee arthralgia was reported by 8 of 11 in the HMDA-HA group, while all others, including both cases in the BDDE-HA group, involved non-target joints (shoulder or wrist). Knee arthralgia AEs were mild to moderate, deemed unrelated to the study intervention or COVID-19, and mostly resolved without the use of rescue medication or additional intervention (Table S10). Based on baseline WBP distributions, Week 12 values, and individual improvement trajectories, knee arthralgia was not considered indicative of insufficient treatment efficacy (Figure S3).

Solicited local AEs at the injection site—primarily pain, warmth, and oedema—were common but transient and mild, with comparable frequencies between groups (90.65% in the HMDA-HA group and 83.33% in the BDDE-HA group), showing no significant inter-group difference (Table 3). No cases of bleeding, induration, redness, or pruritus were reported. Laboratory test abnormalities, changes in vital signs, and physical examination findings were not considered clinically significant or persistent. Although a few electrocardiogram (ECG) changes were noted, none were associated with safety concerns.

## 4. Discussion

This randomised, double-blind, controlled Phase 3 study is the first randomised controlled trial (RCT) in humans to demonstrate that a single-injection intra-articular regimen of the novel HMDA-HA hydrogel is clinically effective and non-inferior to a BDDE-HA hydrogel in reducing WBP at Week 12, with a favourable safety profile, in patients with mild-to-moderate knee OA. The improvement in WBP observed in the HMDA-HA group was mirrored by similar trends across secondary endpoints, including rest, night, and motion pain; patient and investigator global assessments; and WOMAC total and subscale scores. The overall efficacy profile was comparable between groups, supporting the therapeutic non-inferiority of HMDA-HA to a widely used, licensed BDDE-HA-based comparator in alleviating pain and improving function in patients with knee OA.

Both treatments led to statistically and clinically meaningful improvements in pain and function, with effects sustained through Week 36, following a second injection at Week 24. Both groups showed a ≥20 mm reduction in WBP on the 100 mm VAS by Week 6, reaching ≥30 mm after re-dosing at Week 36 (Figure 4a). At that point, over 70% of participants in both groups met the responder criteria (Figure 4a), indicating a substantial and durable clinical benefit of both IAHA products.

Although the difference did not reach statistical significance (*p* = 0.4957), HMDA-HA demonstrated a trend towards enhanced pain relief following re-dosing (Table 2), with a between-group difference of −4.75 mm [95% CI: −10.83 to 1.14] in WBP change at Week 36 relative to the re-dosing baseline at Week 24 (*p* = 0.1129; Table S6). A post-hoc analysis using the minimal clinically important difference (MCID) threshold of 9 mm in WBP [36] showed increasing responder rates over time in both groups (Table S7). Notably, the HMDA-HA group demonstrated a higher responder rate at Week 36 (91.04% vs. 83.52% for BDDE-HA), with a continued upward trajectory (Figure 4b, Table S7). These findings may reflect longer-lasting, clinically meaningful cumulative analgesic effects in favour of HMDA-HA. Unlike BDDE, which crosslinks HA via hydroxyl groups, HMDA crosslinks through carboxyl groups—the primary targets of hyaluronidases—thereby potentially enhancing enzymatic resistance and prolonging intra-articular residence time [13,16].

In physical assessments, HMDA-HA was associated with statistically significant, though not sustained, reductions in joint-line tenderness on pressure at Week 12, as well as sustained trends towards improved range of motion (extension and flexion) (Table 2 and Table S4, Figure S1). These findings may reflect reduced local inflammation and tenderness associated with OA, as previously demonstrated in animal studies [17]. The observed durability of symptom relief and functional improvements may be attributed to distinct structural and physicochemical properties of HMDA-HA conferred by its unique cross-linking mechanism, such as enhanced viscoelasticity and shear viscosity, as described earlier.

From a safety perspective, HMDA is considered as a safer crosslinker, with favourable toxicological profiles and no evidence of reactive residues or toxic metabolites in vivo [15,17]. A full battery of genotoxicity studies of HMDA-HA in bacterial and mammalian systems showed no evidence of mutagenicity or clastogenicity, confirming the absence of genotoxic potential [17]. In the 26-week repeat-dose carcinogenicity study using transgenic mice, the no-observed-adverse-effect levels (NOAELs) of HMDA-HA and HMDA were determined to be 38.52 mg/kg and 46.67 mg/kg, respectively, corresponding to safety margins of approximately 49.5- and 60.0-fold relative to the expected human exposure at the clinical dose [17]. In a 40-week repeat-dose toxicity study in dogs, the estimated safety margin of HMDA-HA ranged from 18.8 to 26.8 [17]. This margin was calculated based on the dog NOAELs, expected intra-articular residence time, exponential decay modelling from non-clinical pharmacokinetic data, and assumptions aligned with a clinical dosing interval of one injection every 6 months.

Consistent with these non-clinical expectations, HMDA-HA was well tolerated throughout the 36-week study period, with no deaths reported (Table 3). No ADRs occurred, in line with incidence ranges reported in previous IAHA studies (0–5.67%) [33,37,38,39,40,41]. While the incidence of treatment-emergent AEs and solicited local injection-site AEs was numerically higher in the HMDA-HA group, most events were mild to moderate and transient, consistent with the known safety profile of IAHA products. Given that the study was conducted during the COVID-19 pandemic, a post-hoc analysis excluding COVID-19 AEs revealed no statistically significant difference in overall AE incidence between groups (9.35% vs. 5.26%; *p* = 0.1034; Table S9). AE incidence did not increase after re-dosing; instead, a ~60% reduction in the number of events was observed in both groups (Tables S11 and S12), indicating no emerging safety concerns following repeated administration.

Although arthralgia was reported more frequently in the HMDA-HA group (10.28% vs. 1.75%; Table 4), the incidence remained within the range previously reported for IAHA studies (4.6–25.2%) [33,37,38,40,41] and most events were self-limiting (Table S10). A post-hoc analysis of WBP score distributions and individual response trajectories indicated that the occurrence of knee arthralgia was sporadic across participants, with no consistent pattern suggestive of treatment failure (Figure S3). The higher incidence in the HMDA-HA group is thus unlikely to be associated with insufficient treatment efficacy. Instead, it may reflect transient joint distension due to a larger injection volume (5 mL vs. 3 mL) or a temporary local tissue response to HMDA-HA. However, volume-related causality appears unlikely, as the incidence of knee arthralgia did not increase following the re-dosing of HMDA-HA (Tables S11 and S12). Furthermore, in the Phase 1/2 study (SP-HA-001), the overall AE incidence was not higher than the 5 mL HMDA-HA group compared with the 3 mL group (one out of eight participants in each group reported an AE), nor was it higher than in the placebo group (three of four participants had five AEs; Table S13). Although antigenicity studies using Hartley guinea pigs and the active systemic anaphylaxis tests or the passive cutaneous anaphylaxis tests did not show antigenic responses to HMDA-HA [17], a mild local tissue reaction cannot be entirely ruled out due to the limited sample size and interspecies difference. Variability in injection techniques—such as procedural accuracy and injector experience—may also influence local tissue response and pain perception, particularly in small-scale studies.

Recent meta-analyses have demonstrated that currently available single-injection cross-linked IAHA products offer efficacy comparable to multiple-dose regimens based on unmodified HA [42]. The above findings support the therapeutic efficacy of HMDA-HA using a single-injection regimen administered at 6-month intervals for knee OA, suggesting it may serve as a viable alternative to conventional multiple-injection IAHA products. Given the advantages of reduced injection frequency, HMDA-HA may help to minimise the risk of synovial tissue damage and infection, as well as patient burden associated with repeated clinical visits.

Several limitations merit consideration. First, although the sample size was adequately powered for the primary endpoint and the study design preserved randomisation by including all participants in the re-dosing phase—thereby avoiding the selection bias typically introduced by responder-only re-injection designs [39]—it may have been underpowered to detect subtle differences in secondary endpoints and subgroup analyses. Second, while the randomised, double-blind design and the use of a clinically relevant active comparator strengthened this study, the study population was limited to Korean patients, which may limit the generalisability of findings to broader, multi-national and multi-ethnic populations. Although subgroup analyses by age suggested potentially consistent efficacy, the limited number of participants aged over 75 years should be noted. Third, although the current study design was mandated by the regulatory authority due to the first-in-human nature of this Phase 3 trial, the relatively short 36-week duration may not adequately capture the long-term efficacy of HMDA-HA, particularly given its potentially prolonged intra-articular residence. With an estimated intra-articular half-life of 123.5 days, longer observation periods—such as 48 to 52 weeks or beyond—would be warranted to better characterise the durability of efficacy and long-term safety profile of HMDA-HA hydrogels following repeated injections. A full-scale, multicentre, randomised trial with extended follow-up would also help more clearly distinguish the sustained therapeutic effects from placebo responses or carry-over effects of prior IAHA or corticosteroid use and address remaining uncertainties regarding repeated dosing. Fourth, the use of another IAHA product as an active comparator may complicate the interpretation of the study results, although BDDE-HA was selected based on ethical and regulatory considerations. While numerous controlled studies have shown that IAHA is comparable in efficacy to systemic interventions such as corticosteroids or NSAIDs [6,43], other studies have raised concerns regarding its limited efficacy and the potential risks associated with viscosupplementation with IAHA [44,45], contributing to conditional recommendations against its routine use for knee OA in guidelines [46,47]. These inconclusive findings may be attributed to the heterogeneity and diversity of IAHA formulations/properties, study designs, patient populations, and contextual factors—including placebo effects—that can confound trial outcomes [48]. Such variability may have also underpowered the present study, despite significant measures to discriminate between true treatment effects and potential sources of bias, such as double-blinding. Although head-to-head comparisons with a placebo such as phosphate buffered saline (PBS) are rarely approved due to ethical constraints, previous placebo-controlled studies (e.g., Gel-200^®^, Seikagaku Corporation, Tokyo, Japan; Adant^®^, Tedec-Meiji Farma, Madrid, Spain) have confirmed the efficacy of IAHA [4,40]. Nevertheless, there remains debate over whether PBS serves as an inert control or exerts a therapeutic effect through a lavage mechanism, such as the dilution and removal of inflammatory mediators, or through the joint aspiration that typically precedes intervention [49]. Lastly, the absence of structural imaging or biomarker assessments (e.g., synovial fluid cytokines and cartilage thickness) limits mechanistic interpretation. Future studies incorporating these endpoints may further elucidate the clinical effects and mechanism of action of HMDA-HA.

## 5. Conclusions

HMDA-HA significantly reduced knee osteoarthritis (OA)-related pain and demonstrated non-inferiority to BDDE-HA at Week 12. This therapeutic effect was sustained over the 36-week study period, with consistent improvements observed across multiple pain and function parameters—and a numerically greater benefit noted following repeated administration. The safety profile of HMDA-HA was favourable, with most adverse events being mild in severity and consistent with those expected for intra-articular therapies. Collectively, these findings support HMDA-HA as a clinically meaningful and well-tolerated treatment option for patients with mild-to-moderate knee OA.

## 6. Patents

The formulation and cross-linking method of HMDA-HA are protected under a Korean registered substance patent (Korea patent No. KR101062320B1), available at https://doi.org/10.8080/1020080074260 (accessed on 16 June 2005). This work is the object of the Shin Poong Pharm. Co., Ltd., patent application (No. 10-2024-0175926).

## Figures and Tables

**Table 1:** Demographics and baseline characteristics (full analysis set).

Variables	HMDA-HA (N = 107)	BDDE-HA (N = 113)	*p* Value [1]
Mean age (SD), years	63.00 (7.53)	63.93 (7.57)	0.3626 (t)
Female, n (%)	81 (75.70)	86 (76.11)	0.9440 (c)
Mean body mass index (SD), kg/m^2^	25.18 (2.80)	24.75 (2.93)	0.2685 (t)
Kellgren & Lawrence grade, n (%)			
Grade I	10 (9.35)	17 (15.04)	0.4245 (c)
Grade II	58 (54.21)	59 (52.21)	
Grade III	39 (36.45)	37 (32.74)	
Mean weight-bearing pain (SD), mm ^†^	55.71 (11.18)	55.77 (12.46)	0.6416 (w)
Mean investigator global assessment (SD), mm ^†^	51.29 (15.80)	52.20 (16.58)	0.7091 (t)
Mean WOMAC total score (SD) ^‡^	44.67 (16.49)	47.29 (15.83)	0.3567 (w)

SD = standard deviation, and WOMAC = Western Ontario and McMaster Universities Osteoarthritis index. N = the number of participants in the specified arm. BMI (kg/m^2^) = weight (kg)/[height/100 (cm)]^2^. [1] Testing for the difference between groups by two-sample *t*-test (t), Wilcoxon rank-sum test (w), or chi-square test (c). ^†^ On a 100 mm visual analogue scale. ^‡^ The WOMAC measures five items for pain (score range 0–20), two items for stiffness (score range 0–8), and 17 items for functional limitation (score range 0–68).

**Table 2:** Key efficacy outcomes (per-protocol set).

Outcome Measures	HMDA-HA (N = 83)	BDDE-HA (N = 95)	LS Mean Difference [95% CI]	*p* Value [1]
Primary outcome *				
Changes from baseline in WBP at Week 12—100 mm VAS, LS mean (SE) mm	−23.72 (1.88)	−25.98 (1.76)	2.26 [−2.83, 7.34]	0.3825
Secondary outcomes **				
Changes from baseline in WBP—100 mm VAS, LS mean (SE) mm				
At Week 2	−11.71 (1.93)	−14.57 (1.80)	2.86 [−2.35, 8.08]	0.2795
At Week 6	−21.75 (1.94)	−22.35 (1.83)	0.60 [−4.66, 5.86]	0.8213
At Week 24	−23.93 (2.08)	−25.90 (2.00)	1.97 [−3.73, 7.67]	0.4957
At Week 36	−32.18 (2.21)	−29.96 (1.90)	−2.22 [−7.98, 3.54]	0.4957
Changes from baseline at Week 12—100 mm VAS, LS mean (SE) mm				
Rest pain	−17.06 (1.85)	−19.32 (1.73)	2.26 [−2.73, 7.26]	0.3717
Night pain	−16.61 (1.99)	−18.43 (1.86)	1.82 [−3.57, 7.21]	0.5054
Motion pain	−22.67 (2.10)	−24.87 (1.96)	2.20 [−3.48, 7.88]	0.4457
Patient global assessment	−23.37 (2.00)	−24.98 (1.87)	1.60 [−3.81, 7.01]	0.5593
Investigator global assessment	−22.54 (1.56)	−25.38 (1.46)	2.84 [−1.39, 7.06]	0.1874
Changes from baseline in WOMAC index at Week 12, LS mean (SE)				
Total score	−15.82 (1.58)	−17.01 (1.48)	1.19 [−3.10, 5.47]	0.5856
Pain subscore	−3.14 (0.34)	−3.58 (0.32)	0.44 [−0.47, 1.35]	0.3429
Function subscore	−11.25 (1.18)	−11.90 (1.10)	0.65 [−2.54, 3.83]	0.6896
Stiffness subscore	−1.47 (0.14)	−1.51 (0.13)	0.04 [−0.35, 0.43]	0.8467
Changes from baseline in physical assessments at Week 12, LS mean (SE)				
Swelling ^†^	−0.20 (0.04)	−0.32 (0.04)	0.12 [0.01, 0.23]	0.0367
Joint-line tenderness on pressure ^‡^	−0.67 (0.06)	−0.50 (0.06)	−0.17 [−0.34, 0.00]	0.0470
Range of motion [extension], degree	−0.24 (0.14)	−0.19 (0.13)	−0.06 [−0.44, 0.32]	0.7672
Range of motion [flexion], degree	−0.55 (0.55)	0.23 (0.52)	−0.78 [−2.27, 0.71]	0.3037
Consumed dose of rescue medication at Week 12, mean (SD) g	5.98 (9.48)	6.07 (10.20)	−0.10 [−3.02, 2.83]	0.4634 (w)

CI = confidence interval, LS mean = least square mean, SD = standard deviation, SE = standard error, VAS = visual analogue scale, and WOMAC = Western Ontario and McMaster Universities Osteoarthritis index. N = the number of participants in the efficacy analysis set in the specified arm. [1] Testing for the difference between treatment groups [analysis of covariance (ANCOVA) model with treatment group as a factor and baseline value as a covariate, or Wilcoxon rank-sum test (w)]. * Change from baseline at Week 12 was calculated by WBP at Week 12 minus WBP at baseline. If the lower limit of the 95% confidence interval for LS mean difference (LS mean of the HMDA-HA group minus LS mean of the BDDE-HA group) is smaller than the non-inferiority margin of 10 mm, HMDA-HA is determined to be non-inferior to BDDE-HA. ** Change from baseline at the designated Week n (n = 2, 6, 12, 24, or 36) was calculated as the designated Week n minus baseline. Between-group differences were calculated as HMDA-HA minus BDDE-HA. † 0 = none (no swelling, absent), 1 = mild (mild reaction on tests such as patella tap test), 2 = moderate (reaction on tests such as patella tap test), and 3 = severe (reaction on tests such as patella tap test, with severe swelling). ‡ 0 = none (no pain), 1 = mild (some pain), 2 = moderate (some pain, with grimacing), and 3 = severe (some pain, with grimacing and withdrawal). ^¶^ Proportion (%) = (The number of participants at each score at Week n)/(The number of participants with available data at Week n in the efficacy analysis set) × 100, n = 2, 6, 12, 24, and 36.

**Table 3:** Overall summary of safety (safety set).

	HMDA-HA (N = 107)	BDDE-HA (N = 114)	*p* Value [1]
AEs ^†^, n (%) [event]	44 (41.12) [84]	32 (28.07) [52]	0.0412 (c)
Mild	32 (29.91) [57]	24 (21.05) [37]	
Moderate	20 (18.69) [26]	11 (9.65) [15]	
Severe	1 (0.93) [1]	0	
SAEs, n (%) [event]	4 (3.74) [4]	3 (2.63) [3]	0.7146 (f)
AEs leading to discontinuation of study intervention, n (%) [event]	1 (0.93) [1]	1 (0.88) [1]	1.0000 (f)
AEs leading to death	0	0	NC
ADRs	0	0	NC
SADRs	0	0	NC
Solicitated local AEs at injection site, n (%) [event]	97 (90.65) [337]	95 (83.33) [307]	0.1072 (c)
Pain	96 (89.72) [161]	95 (83.33) [163]	
Swelling	18 (16.82) [25]	17 (14.91) [20]	
Oedema	38 (35.51) [57]	34 (29.82) [42]	
Erythema	25 (23.36) [32]	21 (18.42) [23]	
Warmth	45 (42.06) [62]	44 (38.60) [59]	

ADRs = adverse drug reactions, AEs = adverse events, SADRs = serious adverse drug reactions, SAEs = serious adverse events, and NC = not calculated. N = the number of participants in the specified arm. The incidence of AEs and ADRs was presented as ‘the number of participants (percentage of participants) [number of events]’ based on the preferred term using MedDRA version 25.1. The denominator for the percentage is the number of participants in each group. [1] Testing for the difference between treatment groups (chi-square test (c) or Fisher’s exact test (f)). ^†^ AEs were collected after obtaining informed consent and listed as treatment-emergent adverse events (TEAEs).

**Table 4:** Incidence of adverse events by system organ class and preferred term (safety set).

	HMDA-HA (N = 107)	BDDE-HA (N = 114)
AEs Occurring in ≥1% of Participants ^†^, n (%) [event]		
Infections and Infestations	15 (14.02) [18]	12 (10.53) [14]
COVID-19	10 (9.35) [10]	6 (5.26) [6]
Urinary tract infection	2 (1.87) [3]	2 (1.75) [2]
Cystitis	2 (1.87) [2]	0
Nasopharyngitis	0	2 (1.75) [2]
Musculoskeletal and Connective Tissue Disorders	16 (14.95) [20]	5 (4.39) [7]
Arthralgia	11 (10.28) [11]	2 (1.75) [2]
Back pain	3 (2.80) [3]	1 (0.88) [1]
Pain in extremity	2 (1.87) [2]	0 (0.00)
Gastrointestinal Disorders	6 (5.61) [8]	4 (3.51) [5]
Investigations	6 (5.61) [10]	2 (1.75) [3]
ALT increased	3 (2.80) [3]	1 (0.88) [1]
AST increased	2 (1.87) [2]	1 (0.88) [1]
Blood glucose increased	2 (1.87) [2]	1 (0.88) [1]
Metabolism and Nutrition Disorders	4 (3.74) [4]	2 (1.75) [2]
Dyslipidaemia	2 (1.87) [2]	0
Eye Disorders	5 (4.67) [6]	0
Conjunctivitis allergic	4 (3.74) [4]	0
Injury, Poisoning and Procedural Complications	2 (1.87) [2]	3 (2.63) [3]
Neoplasms, Benign, Malignant and Unspecified (including Cysts and Polyps)	2 (1.87) [2]	2 (1.75) [2]
Skin and Subcutaneous Tissue Disorders	3 (2.80) [3]	1 (0.88) [1]
Vascular Disorders	2 (1.87) [2]	2 (1.75) [2]
Ear and Labyrinth Disorders	1 (0.93) [1]	2 (1.75) [2]
Vertigo positional	0	2 (1.75) [2]
Renal and Urinary Disorders	1 (0.93) [1]	2 (1.75) [2]
Respiratory, Thoracic and Mediastinal Disorders	0	2 (1.75) [3]
Cardiac Disorders	2 (1.87) [2]	0
Endocrine Disorders	0	2 (1.75) [2]
Thyroid mass	0	2 (1.75) [2]
Nervous System Disorders	2 (1.87) [2]	0

AEs = adverse events, ALT = alanine aminotransferase, AST = aspartate aminotransferase, COVID-19 = coronavirus disease 2019, MedDRA = Medical Dictionary for Regulatory Activities, NC = not calculated, PT = preferred term, and SOC = system organ class. N = the number of participants in the specified arm. The incidence of AEs was displayed as ‘the number of participants (percentage of participants) [number of events]’ based on the preferred term using MedDRA version 25.1. The denominator for the percentage is the number of participants in each group. [1] Testing for the difference between treatment groups (chi-square test (c) or Fisher’s exact test (f)). ^†^ AEs were collected after obtained informed consent as treatment-emergent adverse events (TEAEs).

## Data Availability

The data presented in this study are available upon reasonable request to the corresponding author, subject to written permission from Shin Poong Pharm. Co., Ltd. (Ansan, Republic of Korea). The data are publicly unavailable due to privacy or ethical restriction, and confidentiality.

## Supplementary Materials

The following supporting information can be downloaded at: https://www.mdpi.com/article/10.3390/jcm14124384/s1, Supplementary S1: Supplementary Statistical Analysis; Supplementary S2: Supplementary Data, including the following: Table S1: Inclusion/Exclusion criteria; Table S2: Demographics and baseline characteristics (full analysis set); Table S3: Subgroup analysis of the primary efficacy outcome (per-protocol set; post-hoc analysis); Table S4: Secondary efficacy outcomes for pain and functional improvements (per-protocol set); Table S5: Secondary efficacy outcomes for responder rates (per-protocol set); Table S6: Comparison of WBP changes between Weeks 0–12 and Weeks 24–36 following initial and repeated injections (per-protocol set; post-hoc analysis); Table S7: Responder rates meeting MCID criteria (per-protocol set; post-hoc analysis); Table S8: List of individuals with serious adverse events (safety set); Table S9: Incidence of adverse events excluding COVID-19 (safety set; post-hoc analysis); Table S10: List of individuals with knee arthralgia in the HMDA-HA group (safety set); Table S11: Incidence of adverse events during the 12 weeks following each intra-articular injection (safety set; post-hoc analysis); Table S12: Incidence of adverse events occurring in ≥1% of participants by preferred term (safety set; including post-hoc analysis); Table S13: Summary of overall safety in the 3 mL or 5 mL HMDA-HA groups of the Phase 1/2 study (SP-HA-001) (safety set); Figure S1: Changes from baseline in the range of knee motion through Week 36 (per-protocol set). Figure S2: The total consumed dose of rescue medication through Week 36 (per-protocol set); Figure S3: Changes in WBP over 12 weeks in participants with knee arthralgia in the HMDA group (full analysis set); Supplementary S3: Supplementary Information on the Study Intervention.

## Abbreviations

The following abbreviations are used in this manuscript:

ADR	adverse drug reaction
AE	adverse event
ANCOVA	analysis of covariance
BDDE	1,4-butanedioldiglycidylether
BDDE-HA	1,4-butanedioldiglycidylether cross-linked hyaluronic acid hydrogel
BMI	body mass index
CI	confidence interval
COVID-19	coronavirus disease 2019
DVS	divinyl sulfone
ECG	electrocardiogram
ESCEO	European Society for Clinical and Economic Aspects of Osteoporosis, Osteoarthritis, and Musculoskeletal Diseases
EOS	end-of-study
FAS	full analysis set
GCP	Good Clinical Practice
GMP	Good Manufacturing Practice
HMDA	hexamethylenediamine
HMDA-HA	hexamethylenediamine cross-linked hyaluronic acid hydrogel
Hz	hertz
IAHA	intra-articular hyaluronic acid
ICH	International Council for Harmonisation of technical requirements for pharmaceuticals for human use
IGA	investigator global assessment
IRB	institutional review board
KL grade	Kellgren and Lawrence grade
LOCF	last observation carried forward
LS Mean	least squares mean
MedDRA	Medical Dictionary for Regulatory Activities
NOAEL	no-observed-adverse-effect level
NSAID	non-steroidal anti-inflammatory drug
OA	osteoarthritis
OMERACT-OARSI	Outcome Measures for Rheumatology Committee and Osteoarthritis Research Society International Standing Committee for Clinical Trials Response Criteria Initiative
Pa	pascal
PBS	phosphate buffered saline
PGA	patient global assessment
PPS	per-protocol set
PT	preferred term
RCT	randomised controlled trial
SADR	serious adverse drug reaction
SAE	serious adverse event
SAP	statistical analysis plan
SOC	system organ class
SD	standard deviation
SE	standard error
TCA	tricarboxylic acid
TEAE	treatment emergent adverse event
VAS	visual analogue scale
WBP	weight-bearing pain
WOMAC	Western Ontario and McMaster Universities Osteoarthritis
