# Supplementary material for: A Multicentre, Double-Blind, Randomised, Non-Inferiority Trial of a Novel Single-Injection Intra-Articular HMDA-Cross-Linked Hyaluronate Gel for Knee Osteoarthritis
Source: J Clin Med. 2025 Jun 19;14(12):4384. doi: 10.3390/jcm14124384 (PMC12193865; doi:10.3390/jcm14124384)
Supplement: Supplementary file 1 [file jcm-14-04384-s001.zip › jcm-3662142-supplementary.pdf]

Journal of
Clinical Medicine

MDPI

Supplementary Materials

A Multicentre, Double-Blind, Randomised, Non-Inferiority Trial of a Novel Single-Injection Intra-Articular HMDA-Cross-Linked Hyaluronate Gel for Knee Osteoarthritis

Kang-Il Kim [1,*], Yong In [2], Hyung-Suk Choi [3], Ju-Hong Lee [4], Jae-Ang Sim [5], Han-Jun Lee [6], Young-Wan Moon [7], Oog-Jin Shon [8], Jong-Keun Seon [9], Young-Mo Kim [10], Sang-Jun Song [11], Chong-Bum Chang [12] and Hyuk-Soo Han [13]

Supplementary Materials

Table of Contents

Supplementary S.1. Supplementary Statistical Analysis 1

Supplementary S.2. Supplementary Data 2

Supplementary S.3. Supplementary Information on the Study Intervention 17

References 26

Supplementary S.1. Supplementary Statistical Analysis

The non-inferiority hypothesis was tested as follows:

$$H_0\text{: } \Delta WBP_T - \Delta WBP_C \leq -\delta$$

$$H_1\text{: } \Delta WBP_T - \Delta WBP_C > -\delta$$

where

ΔWBP_T = change in the WBP at Week 12 from baseline of the study drug (HMDA-HA);

ΔWBP_C = change in the WBP at Week 12 from baseline of the control drug (BDDE-HA);

$-\delta$ = non-inferiority margin.

The original statistical analysis plan (SAP) defined the primary endpoint—change in WBP from baseline at Week 12—as [baseline minus Week 12], with a non-inferiority margin of −10 mm. The non-inferiority margin and standard deviation (SD) were based on Ha et al. (2007) [1], which reported a 95% confidence interval (CI) of [−1.9, 10.1] mm for the between-group difference in the WBP change at Week 12. The lower bound of this CI exceeded the −10 mm margin, supporting non-inferiority. Assuming equal SDs in both groups, the pooled SD was calculated to be 22 mm.

To ensure consistency with the direction used for the secondary endpoints and to align with conventions in prior publications, the primary endpoint in this article was redefined as [Week 12 minus baseline], and the non-inferiority margin was adjusted to +10 mm accordingly. The upper bound of the 95% CI of treatment difference—based on adjusted least squares means (HMDA-HA minus BDDE-HA)—was below the +10 mm non-inferiority margin, thereby supporting the non-inferiority of HMDA-HA compared with BDDE-HA. This change affects only the direction of reporting and does not alter the statistical methods, analysis dataset, or interpretation of the results.

Supplementary S.2. Supplementary Data

Table S1. Inclusion/Exclusion criteria.

<table>
<tr><th>Category</th><th>No</th><th>Criteria</th></tr>
<tr><td rowspan="7">Inclusion Criteria</td><td>(1)</td><td>Adult males and females aged 40 years or older as of the date of informed consent.</td></tr>
<tr><td>(2)</td><td>Participant with knee osteoarthritis whose X-ray test results within 6 months from the screening or at the screening visit correspond to grades I–III of the Kellgren & Lawrence scale.
<table><tr><td>Grade 0</td><td>Normal</td></tr><tr><td>Grade I</td><td>Doubtful narrowing of joint space and possible osteophytic lipping</td></tr><tr><td>Grade II</td><td>Definite osteophytes and possible narrowing of joint space</td></tr><tr><td>Grade III</td><td>Moderate multiple osteophytes, definite narrowing of joint space and some sclerosis and possible deformity of bone ends</td></tr><tr><td>Grade IV</td><td>Large osteophytes, marked narrowing of joint space, severe sclerosis and definite deformity of bone ends</td></tr></table></td></tr>
<tr><td>(3)</td><td>Participant diagnosed with unilateral or bilateral knee osteoarthritis according to the clinical diagnostic criteria of the American College of Rheumatology (ACR) at the screening visit who has knee joint pain and meets three or more of the following conditions:
① Over 50 years of age;
② Less than 30 min of morning stiffness;
③ Crepitus on active motion;
④ Bony tenderness;
⑤ Bony enlargement;
⑥ No palpable warmth of synovium.</td></tr>
<tr><td>(4)</td><td>Based on the measurements taken at screening and baseline, weight-bearing pain (WBP) in the affected knee is ≥40 mm as measured by a 100 mm visual analogue scale (VAS).
[However, if both sides have symptoms, the knee joint with the higher WBP becomes the joint for evaluation, and the selected joint cannot be changed during the study period.]</td></tr>
<tr><td>(5)</td><td>Walk without relying on a walking aid such as a walker or cane (patients who have been using a walking aid every day for 6 months prior to the screening visit can use the walking aid, and must use the walking aid in the same way throughout the study period).</td></tr>
<tr><td>(6)</td><td>Ability to fully understand and complete the safety and efficacy measurement questionnaire.</td></tr>
<tr><td>(7)</td><td>Voluntary decision to participate and the provision of informed consent to comply with the precautions after hearing and fully understanding the detailed explanation of the purpose, method, and effects of this study.</td></tr>
<tr><td rowspan="4">Exclusion Criteria</td><td></td><td>[Related to knee osteoarthritis]</td></tr>
<tr><td>(1)</td><td>Body mass index (BMI) ≥35 kg/m^2 at screening.</td></tr>
<tr><td>(2)</td><td>Hip osteoarthritis or osteoarthritis of another joint that is severe enough to preclude the evaluation of knee osteoarthritis at the time of screening.</td></tr>
<tr><td>(3)</td><td>Having the following diseases that may affect the efficacy and safety evaluation, but not being limited to the following:</td></tr>
</table>

Category	No	Criteria
		- Septic arthritis, rheumatoid arthritis, gout, recurrent pseudogout arthritis, trauma that may damage joint cartilage, congenital abnormalities, calcium deposition disease, diabetes, ochronosis, hemochromatosis, acromegaly, Wilson's disease, primary osteochondrosis, genetic diseases (e.g., hyperkinesis), secondary osteoarthritis caused by collagen gene abnormalities, etc.<br>- Sudek's atrophy or severe painful diseases such as Paget's disease.
	(4)	Infection or skin disease in the joint area that is unsuitable for injection at the time of screening.
	(5)	Active or suspected knee joint infection at the time of screening.
	(6)	Complete loss of the patello-femoral joint space.
	(7)	Received the following treatments within 14 days of the screening visit (except those who have passed a 14-day washout period), or the following treatments are scheduled to be administered during the study period:<br>- Medicines containing ingredients such as glucosamine, chondroitin sulphate, and diacerhein.<br>- Herbal medicines and herbal medicines for osteoarthritis.<br>- Anti-inflammatory painkillers, NSAIDs (prescription/non-prescription drugs), etc. (administration of acetaminophen allowed if passing a 3-day washout period).<br>- Oral steroids.<br>- Hospital physical therapy or oriental medicine treatment (cupping, acupuncture, moxibustion, etc.).
	(8)	Intra-articular injection, such as intra-articular corticosteroid, at the injection site within 6 months from the time of screening.
	(9)	Systemic use of steroids within 3 months from the time of screening (including inhalants; however, topical application is permitted if only on the upper body).
	(10)	Hyaluronic acid intra-articular injection administered to the injection site within 6 months from the time of screening.
	(11)	Surgical operations such as knee joint replacement, including arthroscopic surgery, at the administration site within 6 months from the time of screening, or expecting surgery within 10 months.
	(12)	History of artificial joint surgery on the knee joint used for evaluation.
	(13)	Knee joint for evaluation diagnosed as Kellgren Lawrence grade 4 within 6 months from the time of screening.
	(14)	Moderate or severe joint effusion by patella tap test at the screening visit.
		[Medical history]
	(15)	History of hypersensitivity to ingredients of the study intervention (including active control drug).
	(16)	Antiplatelet agents (excluding aspirin of 300 mg/day or less), heparin, oral anticoagulants (coumarin anticoagulants, thrombin inhibitors, Factor Xa inhibitors, etc.), and thrombolytics must be used during the study.
	(17)	Clinically significant abnormalities in liver function (three times more than the upper limit of normal for ALT/AST) or abnormalities in renal function (three times more than the upper limit of normal for serum creatinine), or severe liver or renal disease, at the discretion of the investigator, which is judged to have an impact on the effectiveness and safety evaluation of the study.

Category	No	Criteria
	(18)	The following clinically significant diseases in the past or present:<br>- Diagnosis of malignancy within 5 years prior to screening or currently;<br>- Cardiac disorders (myocardial infarction, coronary artery bypass surgery, arrhythmia, and other serious cardiac disorders, etc.);<br>- Uncontrolled hypertension (systolic blood pressure greater than 160 mmHg or diastolic blood pressure greater than 100 mmHg);<br>- Severe endocrine diseases (diabetes insipidus, Cushing's disease, etc.) which are judged to have an impact on the efficacy and safety evaluation of the study.
		[Others]
	(19)	Currently pregnant or lactating, or planning to become pregnant/lactating during this study.
	(20)	Women with a positive pregnancy test result at the screening visit.
	(21)	Women of childbearing age who have not undergone sterilization surgery, women less than 1 year after menopause, or women who do not agree to avoid pregnancy or use appropriate contraceptive methods* during the study period.<br>*Contraceptive methods: Use (1) surgical sterilization (e.g., bilateral tubal ligation or vasectomy), (2) hormonal agent (implantable, patch, or oral), (3) intrauterine device, or (4) barrier method (male condom, female condom, cervical cap, contraceptive diaphragm, sponge, etc.), or in combination. Combine two or more barrier methods when using spermicide.
	(22)	Planned to participate in another study during the study period or have had (applied) another study intervention (investigational device) within 3 months prior to the screening visit.
	(23)	Difficult to conduct the study as judged by the investigator due to other reasons.

Table S2. Demographics and baseline characteristics (full analysis set).

Variables	HMDA-HA (N = 107)	BDDE-HA (N = 113)	*p* Value [1]
Mean age (SD), years	63.00 (7.53)	63.93 (7.57)	0.3626 (t)
Female, n (%)	81 (75.70)	86 (76.11)	0.9440 (c)
Woman of childbearing potential *, n (%)	5 (6.17)	7 (8.14)	
Mean body mass index (SD), kg/m^2	25.18 (2.80)	24.75 (2.93)	0.2685 (t)
Selected target knee joint, n (%)			0.4073 (c)
Right	48 (44.86)	57 (50.44)	
Kellgren & Lawrence grade, n (%)			0.4245 (c)
Grade I	10 (9.35)	17 (15.04)	
Grade II	58 (54.21)	59 (52.21)	
Grade III	39 (36.45)	37 (32.74)	
Tobacco usage, n (%)	4 (3.74)	1 (0.88)	0.2023 (f)
Alcohol consumption, n (%)	16 (14.95)	12 (10.62)	0.3350 (c)
Mean weight-bearing pain (SD), mm †	55.71 (11.18)	55.77 (12.46)	0.6416 (w)
Mean rest pain (SD), mm †	41.37 (18.17)	43.59 (19.68)	0.4384 (t)
Mean night pain (SD), mm †	44.89 (19.85)	42.31 (22.13)	0.4156 (t)
Mean motion pain (SD), mm †	58.69 (15.45)	59.44 (16.87)	0.7570 (t)
Mean patient global assessment (SD), mm †	54.89 (15.00)	57.32 (16.32)	0.3992 (w)
Mean investigator global assessment (SD), mm †	51.29 (15.80)	52.20 (16.58)	0.7091 (t)
Mean WOMAC total score (SD) ‡	44.67 (16.49)	47.29 (15.83)	0.3567 (w)

**Variables**	**HMDA-HA (N = 107)**	**BDDE-HA (N = 113)**	***p* Value [1]**
Mean WOMAC pain subscore (SD) ‡	8.88 (3.52)	9.47 (3.62)	0.3632 (w)
Mean WOMAC function subscore (SD) ‡	32.00 (11.94)	33.68 (11.44)	0.3775 (w)
Mean WOMAC stiffness subscore (SD) ‡	3.80 (1.68)	4.14 (1.58)	0.1776 (w)
Medical history, n (%)	6 (5.61)	4 (3.54)	NC
Concurrent disease, n (%)	74 (69.16)	92 (81.42)	NC
Prior medication, n (%)	20 (18.69)	18 (15.93)	NC

BMI = body mass index, Max = maximum, Min = minimum, NC = not calculated, SD = standard deviation, and WOMAC = Western Ontario and McMaster Universities Osteoarthritis index. N = the number of participants in the specified arm. BMI (kg/m^2) = weight (kg)/[height/100 (cm)]^2. *The denominator for the percentage is the number of female participants in each group. [1] Testing for the difference between groups by two-sample *t*-test (t), Wilcoxon rank-sum test (w), or chi-square test (c). † On a 100 mm visual analogue scale. ‡ The WOMAC measures five items for pain (score range 0–20), two items for stiffness (score range 0–8), and 17 items for functional limitation (score range 0–68).

Table S3. Subgroup analysis of the primary efficacy outcome (per-protocol set; post-hoc analysis).

**Subgroup**	**Changes from Baseline in WBP at Week 12—100 mm VAS, mm**					***p* Value [1]**
	**HMDA-HA (N = 83)**		**BDDE-HA (N = 95)**		**LS Mean difference [95% CI]**	
	**n**	**LS Mean**	**n**	**LS Mean**		
Overall *	83	−23.72	95	−25.98	2.26 [−2.83, 7.34]	0.3825
Age, years						
≥55	74	−22.72	85	−25.15	2.44 [−2.87, 7.74]	0.3654
<55	9	−29.36	10	−35.37	6.01 [−11.08, 23.10]	0.4670
≥65	30	−27.17	45	−22.58	−4.59 (−12.57, 3.39)	0.2557
<65	53	−21.64	50	−29.18	7.54 [0.90, 14.18]	0.0265
≥75	5	−22.48	9	−24.29	1.80 [−26.45, 30.05]	0.8909
<75	78	−23.80	86	−26.16	2.36 [−2.87, 7.58]	0.3744
Sex						
Male	19	−26.62	23	−25.67	−0.95 [−12.99, 11.09]	0.8740
Female	64	−22.90	72	−26.05	3.16 [−2.54, 8.85]	0.2752
Kellgren & Lawrence grade						
Grade I	7	−22.94	12	−25.45	2.51 [−14.94, 19.95]	0.7644
Grade II	46	−25.41	53	−27.36	1.96 [−4.86, 8.78]	0.5700
Grade III	30	−21.67	30	−23.39	1.72 [−7.40, 10.84]	0.7075

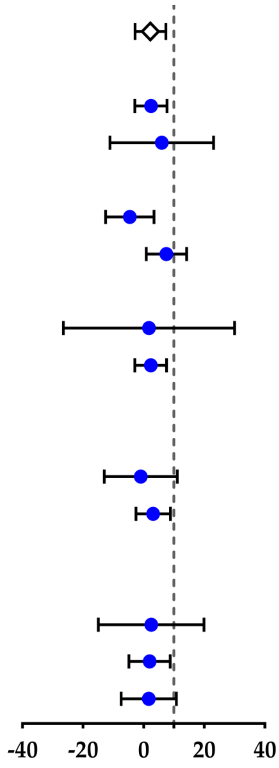

CI = confidence interval, LS mean = least square mean, WBP = weight-bearing pain, and VAS = visual analogue scale. N = the number of participants in the efficacy analysis set in the specified arm. n = the number of evaluable participants. [1] Testing for the difference between treatment groups [analysis of covariance (ANCOVA) model with treatment group as a factor and baseline value as a covariate]. * Change from baseline at Week 12 was calculated by WBP at Week 12 minus WBP at baseline. If the lower limit of the 95% confidence interval for LS mean difference (LS mean of the HMDA-

HA group minus LS mean of the BDDE-HA group) is smaller than the non-inferiority margin of 10 mm, HMDA-HA is determined to be non-inferior to BDDE-HA. The embedded figure presents the between-group LS mean differences (HMDA-HA minus BDDE-HA) with a 95% CI for WBP change from baseline, assessed using a 100 mm VAS. The diamond marker indicates the overall treatment effect; blue circles represent subgroup estimates. The vertical dashed line indicates a non-inferiority margin of 10 mm.

Table S4. Secondary efficacy outcomes for pain and functional improvements (per-protocol set).

Outcome Measures	HMDA-HA (N = 83)	BDDE-HA (N = 95)	Between-Group Difference [95% CI]	*p* Value [1]
Secondary outcomes *				
Changes from baseline in WBP—100 mm VAS, LS mean (SE) mm				
At Week 2	−11.71 (1.93)	−14.57 (1.80)	2.86 [−2.35, 8.08]	0.2795
At Week 6	−21.75 (1.94)	−22.35 (1.83)	0.60 [−4.66, 5.86]	0.8213
At Week 12	−23.72 (1.88)	−25.98 (1.76)	2.26 [−2.83, 7.34]	0.3825
At Week 24	−23.93 (2.08)	−25.90 (2.00)	1.97 [−3.73, 7.67]	0.4957
At Week 36	−32.18 (2.21)	−29.96 (1.90)	−2.22 [−7.98, 3.54]	0.4957
Changes from baseline in rest pain—100 mm VAS, LS mean (SE) mm				
At Week 2	−10.52 (1.76)	−10.28 (1.64)	−0.24 [−5.00, 4.52]	0.9204
At Week 6	−14.09 (1.91)	−16.48 (1.80)	2.38 [−2.79, 7.56]	0.3643
At Week 12	−17.06 (1.85)	−19.32 (1.73)	2.26 [−2.73, 7.26]	0.3717
At Week 24	−16.96 (2.03)	−19.53 (1.94)	2.57 [−2.98, 8.11]	0.3625
At Week 36	−23.71 (1.98)	−24.53 (1.70)	0.82 [−4.35, 5.98]	0.7550
Changes from baseline in night pain—100 mm VAS, LS mean (SE) mm				
At Week 2	−10.34 (1.89)	−8.49 (1.77)	−1.85 [−6.98, 3.28]	0.4768
At Week 6	−13.92 (2.06)	−16.79(1.94)	2.88 [−2.70, 8.46]	0.3101
At Week 12	−16.61 (1.99)	−18.43 (1.86)	1.82 [−3.57, 7.21]	0.5054
At Week 24	−15.87 (2.12)	−18.45 (2.03)	2.58 [−3.24, 8.39]	0.3829
At Week 36	−23.11 (2.02)	−24.04 (1.74)	0.93 [−4.35, 6.21]	0.7284
Changes from baseline in motion pain—100 mm VAS, LS mean (SE) mm				
At Week 2	−11.22 (1.90)	−12.23 (1.78)	1.01 [−4.15, 6.16]	0.7003
At Week 6	−17.40 (2.09)	−22.40 (1.97)	5.00 [−0.68, 10.67]	0.0839
At Week 12	−22.67 (2.10)	−24.87 (1.96)	2.20 [−3.48, 7.88]	0.4457
At Week 24	−18.72 (2.21)	−23.65 (2.12)	4.93 [−1.11, 10.97]	0.1087
At Week 36	−29.49 (2.44)	−31.44 (2.09)	1.95 [−4.40, 8.31]	0.5451
Changes from baseline in patient global assessment100 mm VAS, LS mean (SE) mm				
At Week 2	−13.34 (1.94)	−14.89 (1.82)	1.55 [−3.71, 6.81]	0.5607
At Week 6	−19.22 (2.01)	−21.52 (1.89)	2.31 [−3.16, 7.77]	0.4057

Outcome Measures	HMDA-HA (N = 83)	BDDE-HA (N = 95)	Between-Group Difference [95% CI]	p Value [1]
At Week 12	−23.37 (2.00)	−24.98 (1.87)	1.60 [−3.81, 7.01]	0.5593
At Week 24	−20.42 (2.16)	−23.81 (2.07)	3.39 [−2.52, 9.30]	0.2590
At Week 36	−30.93 (2.16)	−30.23 (1.86)	−0.70 [−6.34, 4.93]	0.8054
Changes from baseline in investigator global assessment—100 mm VAS, LS mean (SE) mm				
At Week 2	−13.50 (1.54)	−14.66 (1.44)	1.16 [−3.01, 5.33]	0.5824
At Week 6	−19.42 (1.71)	−21.16 (1.61)	1.74 [−2.89, 6.36]	0.4600
At Week 12	−22.54 (1.56)	−25.38 (1.46)	2.84 [−1.39, 7.06]	0.1874
At Week 24	−20.16 (1.81)	−22.54 (1.73)	2.38 [−2.56, 7.32]	0.3431
At Week 36	−26.99 (1.87)	−28.32 (1.60)	1.33 [−3.53, 6.20]	0.5892
Changes from baseline in the WOMAC index total score, LS mean (SE)				
At Week 2	−9.87 (1.67)	−10.36 (1.57)	0.49 [−4.05, 5.02]	0.8325
At Week 6	−12.57 (1.66)	−14.07 (1.56)	1.50 [−3.00, 6.00]	0.5102
At Week 12	−15.82 (1.58)	−17.01 (1.48)	1.19 [−3.10, 5.47]	0.5856
At Week 24	−11.92 (1.86)	−14.34 (1.79)	2.41 [−2.68, 7.51]	0.3512
At Week 36	−20.15 (2.01)	−20.23 (1.72)	0.08 [−5.14, 5.30]	0.9756
Changes from baseline in the WOMAC index pain subscore, LS mean (SE)				
At Week 2	−1.98 (0.36)	−2.36 (0.34)	0.38 [−0.60, 1.36]	0.4487
At Week 6	−2.52 (0.37)	−2.55 (0.34)	0.03 [−0.96, 1.02]	0.9498
At Week 12	−3.14 (0.34)	−3.58 (0.32)	0.44 [−0.47, 1.35]	0.3429
At Week 24	−2.42 (0.40)	−2.90 (0.38)	0.49 [−0.61, 1.58]	0.3807
At Week 36	−4.02 (0.42)	−4.27 (0.36)	0.25 [−0.83, 1.34]	0.6452
Changes from baseline in the WOMAC index function subscore, LS mean (SE)				
At Week 2	−6.92 (1.26)	−7.12 (1.18)	0.20 [−3.21, 3.61]	0.9066
At Week 6	−8.76 (1.22)	−10.21 (1.15)	1.46 [−1.85, 4.76]	0.3861
At Week 12	−11.25 (1.18)	−11.90 (1.10)	0.65 [−2.54, 3.83]	0.6896
At Week 24	−8.43 (1.36)	−10.18 (1.31)	1.76 [−1.97, 5.49]	0.3532
At Week 36	−14.55 (1.46)	−14.16 (1.26)	−0.39 [−4.20, 3.42]	0.8411
Changes from baseline in the WOMAC index stiffness subscore, LS Mean (SE)				
At Week 2	−1.03 (0.16)	−0.83 (0.15)	−0.20 (−0.63, 0.23)	0.3511
At Week 6	−1.34 (0.17)	−1.26 (0.16)	−0.07 (−0.54, 0.39)	0.7611
At Week 12	−1.47 (0.14)	−1.51 (0.13)	0.04 (−0.35, 0.43)	0.8467
At Week 24	−1.09 (0.17)	−1.24 (0.17)	0.16 (−0.32, 0.63)	0.5155
At Week 36	−1.62 (0.20)	−1.77 (0.17)	0.15 (−0.37, 0.68)	0.5580
Changes from baseline in swelling †, LS mean (SE)				
At Week 2	−0.28 (0.04)	−0.27 (0.04)	−0.01 (−0.12, 0.10)	0.8475

Outcome Measures	HMDA-HA (N = 83)	BDDE-HA (N = 95)	Between-Group Difference [95% CI]	p Value [1]
At Week 6	−0.30 (0.04)	−0.26 (0.03)	−0.04 (−0.14, 0.06)	0.4461
At Week 12	−0.20 (0.04)	−0.32 (0.04)	0.12 [0.01, 0.23]	0.0367
At Week 24	−0.31 (0.04)	−0.29 (0.04)	−0.02 [−0.12, 0.08]	0.6511
At Week 36	−0.23 (0.04)	−0.33 (0.04)	0.10 [−0.01, 0.20]	0.0817
Changes from baseline in joint-line tenderness on pressure‡, LS mean (SE)				
At Week 2	−0.45 (0.07)	−0.39 (0.07)	−0.06 [−0.26, 0.13]	0.5277
At Week 6	−0.54 (0.07)	−0.42 (0.06)	−0.13 [−0.31, 0.05]	0.1619
At Week 12	−0.67 (0.06)	−0.50 (0.06)	−0.17 [−0.34, 0.00]	0.0470
At Week 24	−0.66 (0.06)	−0.61 (0.05)	−0.05 [−0.21, 0.10]	0.4979
At Week 36	−0.73 (0.06)	−0.65 (0.05)	−0.08 [−0.25, 0.08]	0.3043
Changes from baseline in range of motion (extension), LS mean (SE) degree				
At Week 2	−0.14 (0.17)	0.10 (0.16)	−0.24 [−0.71, 0.22]	0.3042
At Week 6	−0.23 (0.14)	−0.23 (0.13)	0.00 [−0.37, 0.37]	0.9961
At Week 12	−0.24 (0.14)	−0.19 (0.13)	−0.06 [−0.44, 0.32]	0.7672
At Week 24	−0.34 (0.14)	−0.01 (0.13)	−0.33 [−0.71, 0.04]	0.0841
At Week 36	−0.35 (0.15)	−0.28 (0.13)	−0.07 [−0.46, 0.32]	0.7223
Changes from baseline in range of motion (flexion), LS mean (SE) degree				
At Week 2	0.13 (0.61)	−0.09 (0.57)	0.22 [−1.44, 1.88]	0.7950
At Week 6	−0.18 (0.47)	0.15 (0.45)	−0.32 [−1.61, 0.96]	0.6209
At Week 12	−0.55 (0.55)	0.23 (0.52)	−0.78 [−2.27, 0.71]	0.3037
At Week 24	−0.43 (0.64)	−0.17 (0.62)	−0.26 [−2.03, 1.50]	0.7699
At Week 36	−0.79 (0.68)	0.06 (0.58)	−0.85 [−2.62, 0.92]	0.3427
Consumed dose of rescue medication, mean (SD) g				
At Week 2	4.66 (6.02)	3.92 (5.82)	0.74 [−1.02, 2.50]	0.2971(w)
At Week 6	6.02 (10.36)	6.76 (11.21)	−0.39 [−3.95, 2.47]	0.6327 (w)
At Week 12	5.98 (9.48)	6.07 (10.20)	−0.10 [−3.02, 2.83]	0.4634 (w)
At Week 24	6.60 (9.92)	7.75 (11.01)	−1.15 [−4.31, 2.02]	0.8029 (w)
At Week 36	6.13 (8.75)	7.36 (10.75)	−1.23 [−4.33, 1.87]	0.8199 (w)
Proportion of participants taking rescue medicine ¶, %				
At Week 2	60.98	53.68	7.29 [−7.27, 21.85]	0.3285 (c)
At Week 6	50.60	42.11	8.50 [−6.14, 23.13]	0.2565 (c)
At Week 12	49.40	41.05	8.34 [−6.27, 22.96]	0.2642 (c)
At Week 24	48.15	47.83	0.32 [−14.60, 15.24]	0.9663 (c)
At Week 36	56.34	48.35	7.99 [−7.46, 23.43]	0.3128 (c)

CI = confidence interval, LS mean = least square mean, SD = standard deviation, SE = standard error, VAS = visual analogue scale, WOMAC = Western Ontario and McMaster Universities Osteoarthritis index. N = the number of participants in the efficacy analysis set in the specified arm. [1] Testing for the difference between treatment groups (ANCOVA model with treatment group as a factor and

baseline value as a covariate, Wilcoxon rank-sum test (w), or chi-square test (c)). Intra-group differences within each group were tested using either a paired t-test (t) or a Wilcoxon signed-rank test (w), as appropriate. Intra-group differences from baseline within each treatment group were tested using either a paired t-test (t) or a Wilcoxon signed-rank test (w), as appropriate. Statistically significant improvements ($p < 0.0001$) from baseline within each treatment group were observed at all time points in all pain domains, WOMAC indices, and swelling, except for swelling in the HMDA-HA group at Week 12 ($p = 0.0069$) and Week 36 ($p = 0.0019$). *Change from baseline at the designated Week n (n = 2, 6, 12, 24, or 36) was calculated as the designated Week n minus baseline. Between-group differences were calculated as HMDA-HA minus BDDE-HA. †0 = none (no swelling, absent), 1 = mild (mild reaction on tests such as patella tap test), 2 = moderate (reaction on tests such as patella tap test), and 3 = severe (reaction on tests such as patella tap test, with severe swelling). ‡0 = none (no pain), 1 = mild (some pain), 2 = moderate (some pain, with grimacing), and 3 = severe (some pain, with grimacing and withdrawal). ¶ Proportion = (The number of participants at each score at Week n/The number of participants with available data at Week n in the efficacy analysis set) x 100, n = 2, 6, 12, 24, and 36.

Table S5. Secondary efficacy outcomes for responder rates (per-protocol set).

**Outcome Measures**	**HMDA-HA (N = 83)**	**BDDE-HA (N = 95)**	**Odds Ratio [95% CI]**	***p* Value [1]**
Responder rate †, n (%)				
At Week 2	25 (32.47)	34 (38.64)	0.76 [0.40, 1.44]	0.4000
At Week 6	44 (55.70)	55 (61.80)	0.78 [0.42, 1.44]	0.4213
At Week 12	52 (62.65)	63 (66.32)	0.85 [0.46, 1.58]	0.6042
At Week 24	52 (65.00)	56 (64.37)	1.03 [0.55, 1.94]	0.9291
At Week 36	50 (74.63)	66 (72.53)	1.10 [0.53, 2.28]	0.8069
OMERACT-OARSI response rate ‡, n (%)				
At Week 2	34 (44.16)	44 (50.00)	0.79 [0.43, 1.46]	0.4534
At Week 6	43 (54.43)	54 (60.67)	0.77 [0.42, 1.43]	0.4139
At Week 12	52 (62.65)	69 (72.63)	0.63 [0.34, 1.19]	0.1558
At Week 24	46 (57.50)	58 (66.67)	0.68 [0.36, 1.27]	0.2230
At Week 36	49 (73.13)	68 (74.73)	0.92 [0.45, 1.89]	0.8216

CI = confidence interval, OMERACT-OARSI = Outcome Measures for Rheumatology Committee and Osteoarthritis Research Society International Standing Committee for Clinical Trials Response Criteria Initiative, VAS = visual analogue scale, and WOMAC = Western Ontario and McMaster Universities Osteoarthritis index. N = the number of participants in the efficacy analysis set in the specified arm. [1] Testing for the difference between treatment groups (logistic regression model with treatment group as a factor and baseline WBP as a covariate). Responder rate at Week n (%) = (The number of participants with the designated improvements in WBP at Week n/The number of participants with available data at Week n in the efficacy analysis set) x 100, n = 2, 6, 12, 24, and 36. † Responder rate was defined as the proportion of participants with improvement in WBP (%) [with a decrease in WBP measured by a 100 mm VAS of ≥20 mm, or improved by ≥40% from baseline]. ‡ OMERACT-OARSI response rate was defined as the proportion of participants satisfying responder criteria I or II suggested by the OMERACT-OARSI, where criteria I was improvement in WOMAC pain or function ≥50% and absolute change ≥20 points from baseline and criteria II was improvement in at least two of the following three: i. WOMAC pain ≥20% and absolute change ≥10 points from baseline; ii. WOMAC function ≥20% and absolute change ≥10 points from baseline; and iii. patient global assessment (100 mm VAS) ≥20% and absolute change ≥10 mm from baseline.

Table S6. Comparison of WBP changes between Weeks 0–12 and Weeks 24–36 following initial and repeated injections (per-protocol set; post-hoc analysis).

	Changes in WBP for Each Duration —100 mm VAS, mm					*p* Value [1]
	HMDA-HA (N = 83)		BDDE-HA (N = 95)		LS Mean Difference [95% CI]	
	n	LS Mean	n	LS Mean		
12-week duration						
Initial dosing [Week 0–12]	83	−23.72	95	−25.98	2.26 [−2.83, 7.34]	0.3825
Re-dosing [Weeks 24–36]	65	−8.55	86	−3.80	−4.75 [−10.63, 1.14]	0.1129

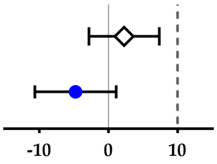

CI = confidence interval, LS mean = least square mean, WBP = weight-bearing pain, and VAS = visual analogue scale. N = the number of participants in the efficacy analysis set in the specified arm. n = the number of evaluable participants. [1] Testing for the difference between treatment groups (ANCOVA model with treatment group as a factor and baseline value as a covariate). *Changes in WBP were calculated as follows: Week 0–12 change = WBP at Week 12 minus baseline; Week 24–36 change = WBP at Week 36 minus WBP at Week 24. The embedded figure displays the LS mean differences (HMDA-HA minus BDDE-HA) in WBP reduction for each period (Weeks 0–12 and Weeks 24–36), along with their corresponding 95% confidence intervals, assessed using a 100 mm VAS. The diamond marker indicates the change observed during Weeks 0–12, while blue circles represent the change during Weeks 24–36. The vertical dashed line denotes the non-inferiority margin of 10 mm.

Table S7. Responder rates meeting MCID criteria (per-protocol set; post-hoc analysis).

Outcome Measures	HMDA-HA (N = 83)	BDDE-HA (N = 95)	Odds Ratio [95% CI]	*p* Value [1]
Responder rate above MCID criteria of 9 mm †, n (%)				
At Week 2	39 (50.65)	53 (60.23)	0.68 [0.37, 1.26]	0.2178
At Week 6	61 (77.22)	68 (76.40)	1.04 [0.50, 2.14]	0.9246
At Week 12	67 (80.72)	76 (80.00)	1.04 [0.49, 2.20]	0.9120
At Week 24	63 (78.75)	73 (83.91)	0.71 [0.32, 1.55]	0.3890
At Week 36	61 (91.04)	76 (83.52)	1.99 [0.72, 5.47]	0.1832

CI = confidence interval, MCID = minimum clinically important difference. N = the number of participants in the efficacy analysis set in the specified arm. [1] Testing for the difference between treatment groups (logistic regression model with treatment group as a factor and baseline WBP as a covariate). Responder rate at Week n (%) = (The number of participants with the designated improvements in WBP at Week n/The number of participants with available data at Week n in the efficacy analysis set) x 100, n = 2, 6, 12, 24, and 36. † Responder rate was defined as the proportion of participants with improvement in WBP (%) [with decrease in WBP measured by 100 mm VAS above the MCID threshold of 9 mm].

Table S8. List of individuals with serious adverse events (safety set).

No.	Study Intervention	Age/ Sex	SAE by PT	Outcome	Intervention Relationship	Start Date (Days from Injection)	AE Duration (Days)
1	HMDA-HA	80/F	Transient cerebral ischemic attack	Recovered/	Not related	84	107

No.	Study Intervention	Age/Sex	SAE by PT	Outcome	Intervention Relationship	Start Date (Days from Injection)	AE Duration (Days)
				resolved		(1st dose)	
2		59/F	Cutaneous horn at the left fifth finger	Recovered/resolved	Not related	109 (1st dose)	21
3		74/F	Bladder tumour	Recovered/resolved	Not related	77 (2nd dose)	5
4		62/F	Angiography and embolization due to pulmonary arteriovenous malformation	Recovered/resolved	Not related	92 (1st dose)	2
5	BDDE-HA	62/F	Right knee degenerative meniscus tear	Recovered/resolved	Not related	4 (1st dose)	24
6		62/F	Pneumonia	Recovered/resolved	Not related	34 (1st dose)	32
7		58/F	Fracture of metatarsal bone, closed	Recovered/resolved	Not related	52 (2nd dose)	34

F = female, AE = adverse event, PT = preferred term, and SAE = serious adverse event.

Table S9. Incidence of adverse events excluding COVID-19 (safety set; post-hoc analysis).

	HMDA-HA (N = 107)	BDDE-HA (N = 114)	*p* Value [1]
AEs †, n (%) [event]	44 (41.12) [84]	32 (28.07) [52]	0.0412 (c)
AEs excluding COVID-19 ‡, n (%) [event]	38 (35.51) [74]	29 (25.44) [46]	0.1034 (c)
AEs occurring in ≥1% of participants excluding COVID-19 ‡, n (%) [event]			
Infections and Infestations	5 (4.67) [8]	7 (6.14) [8]	
Urinary tract infection	2 (1.87) [3]	2 (1.75) [2]	
Cystitis	2 (1.87) [2]	0	
Nasopharyngitis	0	2 (1.75) [2]	
Musculoskeletal and Connective Tissue Disorders	16 (14.95) [20]	5 (4.39) [7]	
Arthralgia	11 (10.28) [11]	2 (1.75) [2]	
Back pain	3 (2.80) [3]	1 (0.88) [1]	
Pain in extremity	2 (1.87) [2]	0 (0.00)	
Gastrointestinal Disorders	6 (5.61) [8]	4 (3.51) [5]	
Investigations	6 (5.61) [10]	2 (1.75) [3]	
ALT increased	3 (2.80) [3]	1 (0.88) [1]	
AST increased	2 (1.87) [2]	1 (0.88) [1]	
Blood glucose increased	2 (1.87) [2]	1 (0.88) [1]	
Metabolism and Nutrition Disorders	4 (3.74) [4]	2 (1.75) [2]	
Dyslipidaemia	2 (1.87) [2]	0	
Eye Disorders	5 (4.67) [6]	0	
Conjunctivitis allergic	4 (3.74) [4]	0	
Injury, Poisoning and Procedural Complications	2 (1.87) [2]	3 (2.63) [3]	
Neoplasms, Benign, Malignant and Unspecified (including Cysts and Polyps)	2 (1.87) [2]	2 (1.75) [2]	
Skin and Subcutaneous Tissue Disorders	3 (2.80) [3]	1 (0.88) [1]	
Vascular Disorders	2 (1.87) [2]	2 (1.75) [2]	
Ear and Labyrinth Disorders	1 (0.93) [1]	2 (1.75) [2]	

Vertigo positional	0	2 (1.75) [2]
Renal and Urinary Disorders	1 (0.93) [1]	2 (1.75) [2]
Respiratory, Thoracic and Mediastinal Disorders	0	2 (1.75) [3]
Cardiac Disorders	2 (1.87) [2]	0
Endocrine Disorders	0	2 (1.75) [2]
Thyroid mass	0	2 (1.75) [2]
Nervous System Disorders	2 (1.87) [2]	0

AEs = adverse events, ALT = alanine aminotransferase, AST = aspartate aminotransferase, COVID-19 = coronavirus disease 2019, MedDRA = Medical Dictionary for Regulatory Activities, NC = not calculated, PT = preferred term, and SOC = system organ class. N = the number of participants in the specified arm. The incidence of AEs was presented as 'the number of participants (percentage of participants) [number of events]' based on the preferred term using MedDRA version 25.1. The denominator for the percentage is the number of participants in each group. [1] Testing for the difference between treatment groups (chi-square test (c) or Fisher's exact test (f)). † AEs were collected after obtaining informed consent and listed as treatment-emergent adverse events (TEAEs). ‡ A conservative approach was applied for the post-hoc analysis of AEs excluding COVID-19. Participants who reported COVID-19 infection as the sole AE (six in the HMDA-HA group vs. three in the BDDE-HA group) and those with symptoms occurring during the infection period that were deemed COVID-19–related by the investigator were excluded. As no further COVID-19–related AEs were identified beyond the confirmed COVID-19 cases, no additional exclusions were made.

Table S10. List of individuals with knee arthralgia in the HMDA-HA group (safety set).

No	Age/ Sex	Severity	Intervention Relationship	AE Treatment	Outcome	Start Date (Days from Injection)	AE Duration (Days)	COVID-19 Infection Status
1	63/F	Mild	Not related	None	Recovered/resolved	87 (1st dose)	Ongoing	No
2	68/F	Moderate	Not related	None	Recovered/resolved	16 (1st dose)	51	No
3	62/F	Mild	Not related	None	Recovered/resolved	14 (1st dose)	26	No
4	73/F	Mild	Not related	None	Recovered/resolved	23 (1st dose)	41	No
5	69/F	Moderate	Not related	Acetaminophen (9.5 g)	Recovered/resolved	9 (2nd dose)	31	No
6	66/F	Moderate	Not related	Acetaminophen (5.0 g)	Ongoing	5 (2nd dose)	Drop-out	No
7	67/F	Mild	Not related	Acetaminophen (3.0 g)	Recovered/resolved	14 (1st dose)	20	No
8	66/F	Mild	Not related	Acetaminophen (3.0 g)	Recovered/resolved	9 (1st dose)	20	No

AE = adverse event; F = female.

Table S11. Incidence of adverse events during the 12 weeks following each intra-articular injection (safety set; post-hoc analysis).

	Incidence During the First 12 Weeks After Initial Dosing [Week 0–12]		Incidence During the First 12 Weeks After Re-dosing [Week 24–36]	
	**HMDA-HA (N = 107)**	**BDDE-HA (N = 114)**	**HMDA-HA (N = 90)**	**BDDE-HA (N = 103)**
Participants with AEs†, n (%) [number of events]	23 (21.50) [38]	17 (14.91) [23]	14 (15.56) [15]	9 (8.74) [9]
Within-group comparison vs. first injection, *p* value			0.0719 (g)	0.0881 (g)
Between-group comparison, *p* value	0.2040 (c)	-	0.1447 (c)	-

	Incidence During the First 12 Weeks After Initial Dosing [Week 0–12]		Incidence During the First 12 Weeks After Re-dosing [Week 24–36]	
	HMDA-HA (N = 107)	BDDE-HA (N = 114)	HMDA-HA (N = 90)	BDDE-HA (N = 103)
Solicited local injection site AEs, n (%) [number of events]	93 (86.92) [215]	90 (78.95) [191]	59 (65.56) [122]	63 (61.17) [116]
Within-group comparison vs. first injection, *p* value			<0.0001	<0.0001
Between-group comparison, *p* value	0.1167 (c)	-	0.5280 (c)	-

AEs = adverse events. N = the number of evaluable participants in the specified arm. The incidence of AEs was presented as 'the number of participants (percentage of participants) [number of events]'. The denominator for the percentage is the number of participants in each group. Adverse events (AEs) were evaluated separately during the 12 weeks following the initial injection (Week 0 to Week 12) and re-injection (Week 24 to Week 36). Within-group comparisons were conducted to assess changes in AE incidence after re-injection versus initial injection. [1] Testing for the difference within treatment groups (generalized estimating equation method (g)) and between treatment groups (chi-square test (c) or Fisher's exact test (f)). † AEs were collected after obtaining informed consent and listed as treatment-emergent adverse events (TEAEs).

Table S12. Incidence of adverse events occurring in ≥1% of participants by preferred term (safety set; including post-hoc analysis).

	Incidence over 36 Weeks After Two Doses [Week 0–36]		Incidence During the First 12 Weeks After Initial Dosing [Week 0–12]		Incidence During the First 12 Weeks After Re-dosing [Week 24–36]	
	HMDA-HA (N = 107)	BDDE-HA (N = 114)	HMDA-HA (N = 107)	BDDE-HA (N = 114)	HMDA-HA (N = 90)	BDDE-HA (N = 103)
AEs †, n (%) [event]						
Infections and Infestations	15 (14.02) [18]	12 (10.53) [14]	6 (5.61) [7]	6 (5.26) [7]	5 (5.56) [5]	3 (2.91) [3]
COVID-19	10 (9.35) [10]	6 (5.26) [6]	2 (1.87) [2]	2 (1.75) [2]	3 (3.33) [3]	2 (1.94) [2]
Urinary tract infection	2 (1.87) [3]	2 (1.75) [2]	1 (0.93) [1]	1 (0.88) [1]	2 (2.22) [2]	0
Cystitis	2 (1.87) [2]	0	2 (1.87) [2]	0	0	0
Nasopharyngitis	0	2 (1.75) [2]	0	1 (0.88) [1]	0	0
Musculoskeletal and Connective Tissue Disorders	16 (14.95) [20]	5 (4.39) [7]	10 (9.35) [13]	1 (0.88) [2]	3 (3.33) [3]	1 (0.97) [1]
Arthralgia	11 (10.28) [11]	2 (1.75) [2]	7 (6.54) [7]	1 (0.88) [1]	2 (2.22) [2]	0
Back pain	3 (2.80) [3]	1 (0.88) [1]	1 (0.93) [1]	0	1 (1.11) [1]	0
Pain in extremity	2 (1.87) [2]	0	2 (1.87) [2]	0	0	0
Gastrointestinal Disorders	6 (5.61) [8]	4 (3.51) [5]	2 (1.87) [2]	2 (1.75) [2]	1 (1.11) [1]	1 (0.97) [1]
Constipation	1 (0.93) [2]	1 (0.88) [1]	1 (0.93) [1]	1 (0.88) [1]	1 (1.11) [1]	0 (0.00) [0]
Investigations	6(5.61) [10]	2(1.75) [3]	4 (3.74) [7]	1 (0.88) [1]	0	0
ALT increased	3 (2.80) [3]	1 (0.88) [1]	2 (1.87) [2]	0	0	0
AST increased	2 (1.87) [2]	1 (0.88) [1]	2 (1.87) [2]	0	0	0
Blood glucose increased	2 (1.87) [2]	1 (0.88) [1]	2 (1.87) [2]	1 (0.88) [1]	0	0

	Incidence over 36 Weeks After Two Doses [Week 0–36]		Incidence During the First 12 Weeks After Initial Dosing [Week 0–12]		Incidence During the First 12 Weeks After Re-dosing [Week 24–36]	
	HMDA-HA (N = 107)	BDDE-HA (N = 114)	HMDA-HA (N = 107)	BDDE-HA (N = 114)	HMDA-HA (N = 90)	BDDE-HA (N = 103)
Metabolism and Nutrition Disorders	4 (3.74) [4]	2 (1.75) [2]	0	1 (0.88) [1]	1 (1.11) [1]	1 (0.97) [1]
Dyslipidaemia	2 (1.87) [2]	0	0	0	1 (1.11) [1]	0
Eye Disorders	5(4.67) [6]	0	3 (2.80) [4]	0	1 (1.11) [1]	0
Conjunctivitis allergic	4 (3.74) [4]	0	3 (2.80) [3]	0	1 (1.11) [1]	0
Neoplasms, Benign, Malignant and Unspecified (including Cysts and Polyps)	2 (1.87) [2]	2 (1.75) [2]	0	1 (0.88) [1]	1 (1.11) [1]	1 (0.97) [1]
Bladder neoplasm	1 (0.93) [1]	0	0	0	1 (1.11) [1]	0
Skin and Subcutaneous Tissue Disorders	3 (2.80) [3]	1 (0.88) [1]	1 (0.93) [1]	0	1 (1.11) [1]	0
Erythema	1 (0.93) [1]	0	0	0	1 (1.11) [1]	0
Vascular Disorders	2 (1.87) [2]	2 (1.75) [2]	1 (0.93) [1]	1 (0.88) [1]	0	0
Hypertension	2 (1.87) [2]	1 (0.88) [1]	1 (0.93) [1]	0	0	0
Ear and Labyrinth Disorders	1 (0.93) [1]	2 (1.75) [2]	0	1 (0.88) [1]	0	0
Vertigo positional	0	2 (1.75) [2]	0	1 (0.88) [1]	0	0
Cardiac Disorders	2 (1.87) [2]	0	0	0	1 (1.11) [1]	0
Angina pectoris	1 (0.93) [1]	0	0	0	1 (1.11) [1]	0
Endocrine Disorders	0	2 (1.75) [2]	0	1 (0.88) [1]	0	0
Thyroid mass	0	2 (1.75) [2]	0	1 (0.88) [1]	0	0
General Disorders and Administration Site Conditions	1 (0.93) [1]	1 (0.88) [1]	0	0	1 (1.11) [1]	0
Peripheral swelling	(0.93) [1]	0	0	0	1 (1.11) [1]	0

AEs = adverse events, ALT = alanine aminotransferase, AST = aspartate aminotransferase, COVID-19 = coronavirus disease 2019, MedDRA = Medical Dictionary for Regulatory Activities, NC = not calculated, PT = preferred term, and SOC = system organ class. N = the number of participants in the specified arm. The incidence of AEs was displayed as 'the number of participants (percentage of participants) [number of events]' based on the preferred term using MedDRA version 25.1. The denominator for the percentage is the number of participants in each group. † AEs were collected after obtaining informed consent and listed as treatment-emergent adverse events (TEAEs).

Table S13. Summary of overall safety in the 3 mL or 5 mL HMDA-HA groups of the Phase 1/2 study (SP-HA-001) (safety set).

	Cohort A		Cohort B	
	HMDA-HA 3 mL (N = 8)	Placebo (3 mL) (N = 2)	HMDA-HA 5 mL (N = 8)	Placebo (5 mL) (N = 2)
AEs †, n (%) [event]	1 (12.5) [1]	1 (50.0) [1]	1 (12.5) [1]	2 (100.0) [4]
Mild	1 (12.5) [1]	0	0	1 (50.0) [3]

	Cohort A		Cohort B	
	**HMDA-HA 3 mL (N = 8)**	**Placebo (3 mL) (N = 2)**	**HMDA-HA 5 mL (N = 8)**	**Placebo (5 mL) (N = 2)**
Moderate	0	1 (50.0) [1]	0	1 (50.0) [1]
Severe	0	0	1 (12.5) [1]	0
SAEs, n (%) [event]	0	1 (50.0) [1]	0	0
AEs leading to discontinuation of study intervention, n (%) [event]	0	0	0	0
AEs leading to death	0	0	0	0
ADRs	0	0	0	0
SADRs	0	0	0	0
Solicitated local AEs at injection site‡, n (%) [event]	0	0	0	0

ADRs = adverse drug reactions, AEs = adverse events, SADRs = serious adverse drug reactions, and SAEs = serious adverse events. N = the number of participants in the specified arm. Note: Patients with knee osteoarthritis (OA) were randomized to receive a single intra-articular injection of HMDA-HA: 3 mL (Cohort A), 5 mL (Cohort B), or an equivalent volume of placebo (saline) at baseline (Week 0), with follow-up through Week 24. The safety analysis set included all randomized participants who received at least one dose of the study intervention. † AEs were collected after obtaining informed consent and listed as treatment-emergent adverse events (TEAEs). In Cohort A, one participant in each group experienced an AE. In the 3 mL HMDA-HA group, one participant reported mild visual acuity reduction (age-related) and withdrew consent. In the placebo group, one participant experienced a moderate SAE (lumbar vertebral fracture) after Week 6, required hospitalization, and discontinued the study. In Cohort B, one participant in the 5 mL HMDA-HA group reported a severe, treatment-unrelated AE (back pain) and withdrew at Week 0. In the placebo group, one participant had a moderate AE (alopecia), and another experienced three mild AEs (hepatic cyst, steatosis, and diabetes mellitus), withdrawing after Week 12. All reported AEs were considered unrelated to the study intervention. ‡Solicitated local AEs at the injection site included swelling, erythema, pain, haematoma, and papule.

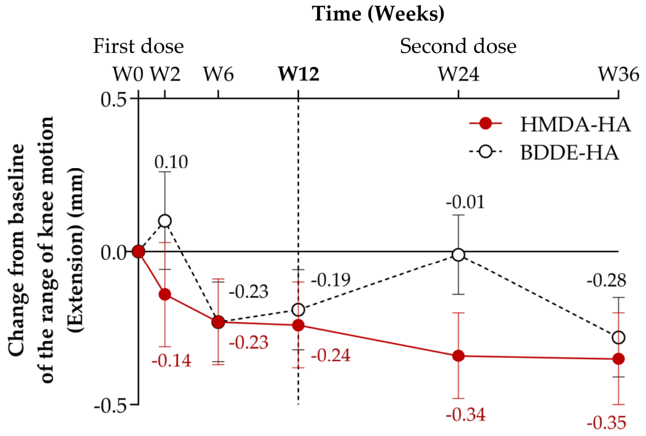

(**a**)

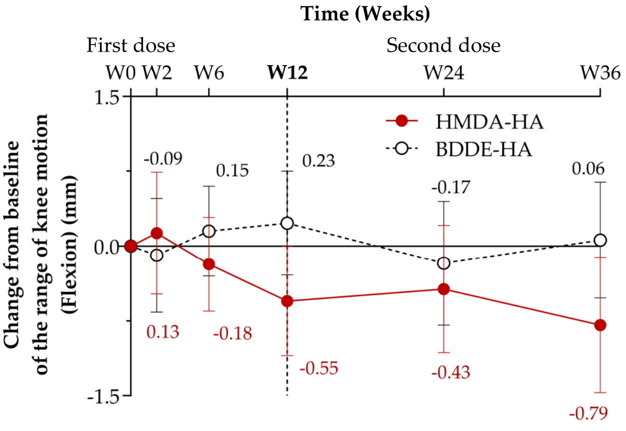

(**b**)

Figure S1. Changes from baseline in the range of knee motion through Week 36 (per-protocol set). Participants received intra-articular injections at baseline (Week 0) and again at Week 24. The graph and error bars represent least squares mean (LS mean) changes and standard errors (SEs) in in the range of motion for the HMDA-HA group (red solid line) and the BDDE-HA group (black dashed

line). Data labels indicate LS mean change in degrees at each time point. No significant differences were observed between groups at any visit by an analysis of covariance. The vertical dashed line indicates the primary endpoint time point (Week 12). (**a**) Extension. (**b**) Flexion.

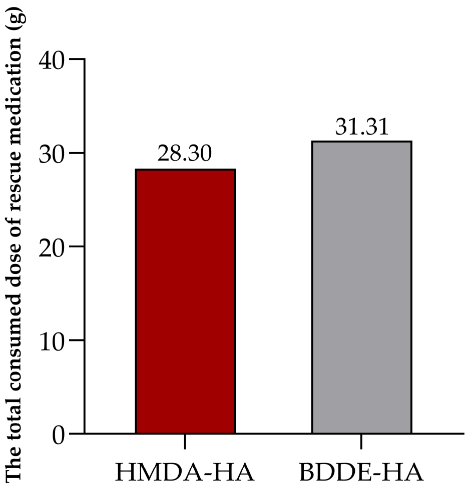

Figure S2. The total consumed dose of rescue medication through Week 36 (per-protocol set). Participants received intra-articular injections at baseline (Week 0) and again at Week 24. The graph represents the total consumed dose of rescue medication in grams (g).

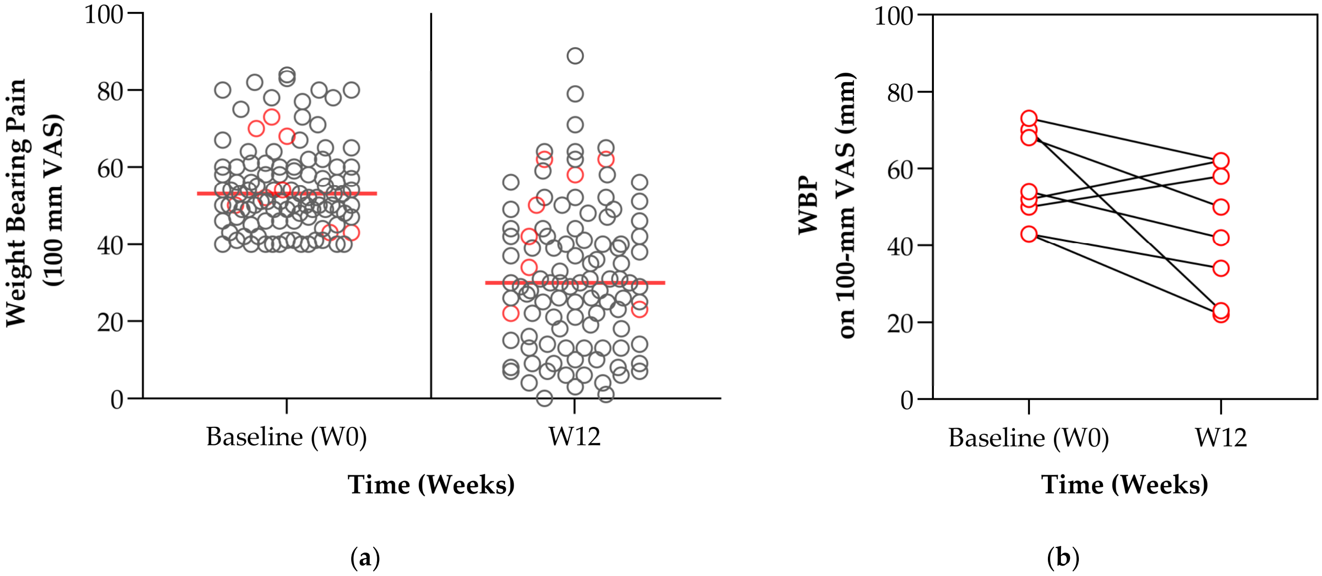

Figure S3. Changes in WBP over 12 weeks in participants with knee arthralgia in the HMDA group (full analysis set). Participants received a single intra-articular injection at baseline (Week 0, W0). (**a**) Distribution of WBP at baseline and Week 12 in all participants (black circles, n = 107), with those experiencing knee arthralgia adverse events highlighted (red circles, n = 7) in the HMDA-HA group. Horizontal red bars indicate the median WBP values for the HMDA-group at each time point. (**b**) Individual WBP trajectories from baseline (before treatment) to Week 12 (post-treatment) in participants with knee arthralgia. Red circles represent WBP values, and connecting lines depict within-subject changes.

Supplementary S.3. Supplementary Information on the Study Intervention

The following table is an excerpt from the Product Overview and Prescribing Information for the HMDA-HA hydrogel [code: SP5M001; brand name: Hyalflex®; 5 mL per prefilled syringe containing 100 mg of sodium hyaluronate cross-linked with hexamethylenediamine (HMDA), manufactured by Shin Poong Pharm. Co., Ltd. (Ansan, Republic of Korea)] (originally in Korean) [2].

Please note that the official version of this document is written in Korean. This English translation is provided for reference only. For any matters of legal or regulatory importance, please consult the original Korean text [available online: https://nedrug.mfds.go.kr/pbp/CCBBB01/getItemDetailCache?cacheSeq=202401722 (accessed on 3 June 2025)].

Original Text [In Korean]	Translated Text [In English]
사용상의 주의사항	**Precautions for Use**
1. 다음 환자에는 투여하지 말 것<br>(1) 이 약 및 이 약의 구성성분에 대해 과민증의 병력이 있는 환자<br>(2) 투여 관절강에 감염 또는 심한 염증이 있는 환자<br>(3) 투여부위의 피부에 감염 또는 피부질환이 있는 환자	1. Contraindications. This product must not be administered to the following patients:<br>(1) Patients with a known hypersensitivity to this product or any of its components.<br>(2) Patients with infections or severe inflammation in the joint to be treated.<br>(3) Patients with infections or dermatologic conditions at the injection site.
2. 다음 환자에는 신중히 투여할 것<br>1) 다른 약물에 대해 과민증의 병력이 있는 환자<br>2) 간장애 또는 그 병력이 있는 환자	2. Warnings and Precautions. Administer with caution in the following patients:<br>(1) Patients with a history of hypersensitivity to other drugs.<br>(2) Patients with liver dysfunction or a history thereof.
3. 이상반응<br>(1) 임상시험은 다양한 조건에서 수행되므로, 특정 임상시험에서 관찰된 이상반응 발현율은 다른 임상시험에서 관찰된 이상반응 발현율과 직접적으로 비교될 수 없으며, 실제 관찰되는 비율을 반영하지 않을 수 있다.<br>(2) 무릎골관절염 환자 대상 시노비안주를 활성대조군으로 한 이 약의 임상시험(SP-HA-003)에 등록된 223 명 의 시험대상자 중 221 명에서 이 약 또는 시노비안주를 관절강 내 1 차 투여 및 24 주차 재투여 받은 후 36 주차까지 보고된 주사부위 국소이상반응의 발현빈도는 이 약 투여군에서 90.65% (97/107 명), 시노비안주 투여군에 서 83.33% (95/114 명)이었으며, 보고된 주사부위 국소이상반응은 아래 표 1 과 같다.	3. Adverse Events<br>(1) As clinical studies are conducted under varying conditions, the incidence rates of adverse reactions observed in one clinical study cannot be directly compared with those in other studies and may not reflect the actual incidence rates.<br>(2) Among the 223 patients with knee osteoarthritis enrolled in the clinical study (SP-HA-003) comparing this product with an active comparator (Synovian injection), 221 received intra-articular injections of either this product or Synovian at baseline and again at Week 24, and the incidence of solicited local adverse events (AEs) at the injection site reported through Week 36 was 90.65% (97/107) in the group treated with this product and 83.33% (95/114) in the group treated with Synovian. The reported solicited local AEs at the injection site are summarized in Table 1.

Original Text [In Korean]	Translated Text [In English]
사용상의 주의사항	**Precautions for Use**

표 1. 이 약의 임상시험(SP-HA-003)에서 보고된 주사부위 국소이상반응

	이 약 투여군 (107 명) n (%)	시노비안주 투여군 (114 명) n (%)
통증	96 (89.72)	95 (83.33)
열감	45 (42.06)	44 (38.60)
부종	38 (35.51)	34 (29.82)
홍반	25 (23.36)	21 (18.42)
종창	18 (16.82)	17 (14.91)

Table 1. Solicited local AEs at the injection site reported in the clinical study of this product (SP-HA-003).

	This product group (N = 107) n (%)	Synovian group (N = 114) n (%)
Pain	96 (89.72)	95 (83.33)
Warmth	45 (42.06)	44 (38.60)
Oedema	38 (35.51)	34 (29.82)
Erythema	25 (23.36)	21 (18.42)
Swelling	18 (16.82)	17 (14.91)

이 약 투여 후 7 일 이상 지속된 주사부위 국소이상반응은 이 약 투여군에서 통증(57.94%), 부종(14.02%), 열감(8.41%), 종창(3.74%), 홍반(3.74%)이었고, 시노비안주 투여군에서는 통증(53.51%), 부종(9.65%), 열감(6.14%), 종창(2.63%), 홍반(2.63%) 순으로 보고되었다. 상기한 주사부위 국소이상반응들은 이 약 투여군 59.79% (58/97 명), 시노비안주 투여군 67.37% (64/95 명)에서 14 일 이내 소실되었고, 대부분 허용된 구제약물만 복용하였거나 특별한 처치 없이 소실되었으며, 이 약 투여군 80.41%, 시노비안주 투여군 82.11%에서 45 일 이내에 소실되었다. 중대한 주사부위 국소이상반응은 보고되지 않았으며, 이 약 투여 후 중증 주사부위 국소이상반응은 통증(29.91%), 부종(3.74%), 열감(0.93%), 종창(0.93%), 홍반(0.93%) 순으로 보고되었다.

Solicited local AEs at the injection site lasting more than 7 days after the administration of this product were reported as pain (57.94%), oedema (14.02%), warmth (8.41%), swelling (3.74%), and erythema (3.74%) in the group treated with this product, and as pain (53.51%), oedema (9.65%), warmth (6.14%), swelling (2.63%), and erythema (2.63%) in the group treated with Synovian; these local AEs resolved within 14 days in 59.79% (58/97) of patients in the group treated with this product and 67.37% (64/95) in the Synovian group, mostly with the use of permitted rescue medications or without any specific treatment, and resolved within 45 days in 80.41% and 82.11% of patients in the respective groups, with no serious local AEs at the injection site reported and severe local AEs at the injection site following this product administration being reported as pain (29.91%), oedema (3.74%), warmth (0.93%), swelling (0.93%), and erythema (0.93%).

이 약을 관절강 내 1 차 투여 후 12 주 동안 또는 재투여 후 같은 기간 동안 보고된 주사부위 국소이상반응의 발현 빈도는 이 약 투여군에서 86.92% (93/107 명), 이 약 재투여 후 65.56% (59/90 명)이었으며, 1 차 투여 또는 재투여 후 보고된 주사부위 국소이상반응은 아래 표 2 와 같다.

The incidence of solicited local AEs at the injection site reported within 12 weeks after the initial or repeat intra-articular administration of this product was 86.92% (93/107) following the initial injection and 65.56% (59/90) following the repeat injection, with the reported reactions summarised in Table 2.

표 2. 이 약의 임상시험(SP-HA-003)에서 1 차 투여 또는 재투여 후 12 주 동안 보고된 주사부위 국소이상반응

Table 2: Solicited local AEs at the injection site reported within 12 weeks following initial or repeated administration in the clinical study of this product (SP-HA-003).

Group	Initial dosing	Re-dosing

Original Text [In Korean]	Translated Text [In English]
사용상의 주의사항	Precautions for Use

구분	이 약 1 차 투여 후 (107 명) n (%)	이 약 재투여 후 (90 명) n (%)
통증	93 (86.92)	58 (64.44)
열감	39 (36.45)	19 (21.11)
부종	33 (30.84)	22 (24.44)
홍반	21 (19.63)	10 (11.11)
종창	17 (15.89)	8 (8.89)

	(N = 107) n (%)	(N = 90) n (%)
Pain	93 (86.92)	58 (64.44)
Warmth	39 (36.45)	19 (21.11)
Oedema	33 (30.84)	22 (24.44)
Erythema	21 (19.63)	10 (11.11)
Swelling	17 (15.89)	8 (8.89)

(3) 무릎골관절염 환자 대상 시노비안주를 활성대조군으로 한 이 약의 임상시험(SP-HA-003)에 등록된 223 명 의 시험대상자 중 221 명에서 이 약 또는 시노비안주를 관절강 내 1 차 투여 및 24 주차 재투여 받은 후 36 주차까 지 보고된 이상반응(Treatment-Emergent Adverse Events, TEAEs) 발현빈도는 이 약 투여군에서 41.12% (44/107 명), 시노비안주 투여군에서 28.07% (32/114 명) 이었으며, 대부분 경증에서 중등증이었다. 이 약 또 는 시노비안주 투여 후 1% 이상의 발현빈도로 보고된 이상반응은 아래 표 3 과 같다.

(3) Among the 223 patients with knee osteoarthritis enrolled in the clinical study (SP-HA-003) comparing this product with an active comparator (Synovian injection), 221 received intra-articular injections of either this product or Synovian at baseline and again at Week 24, and the incidence of treatment-emergent adverse events (TEAEs) up to Week 36 were reported in 41.12% (44/107) of the treatment group and 28.07% (32/114) of the comparator group. Most events were mild to moderate. Table 3 lists AEs reported in ≥1% of participants following the administration of this product or Synovian injection.

표 3. 이 약의 임상시험(SP-HA-003)에서 1% 이상 보고된 이상반응(TEAEs)

구분	이 약 투여군 (107 명) n (%)	시노비안주투여군 (114 명) n (%)
관절통	11 (10.28)	2 (1.75)
COVID-19	10 (9.35)	6 (5.26)
알레르기 결막염	4 (3.74)	0 (0.00)
요로 감염	2 (1.87)	2 (1.75)
알라닌 아미노전이효소 증가	3 (2.80)	1 (0.88)
등허리 통증	3 (2.80)	1 (0.88)
아스파르트산 아미노전이효소 증가	2 (1.87)	1 (0.88)
혈당 증가	2 (1.87)	1 (0.88)
사지 통증	2 (1.87)	0 (0.00)
방광염	2 (1.87)	0 (0.00)
이상 지질 혈증	2 (1.87)	0 (0.00)
고혈압	2 (1.87)	1 (0.88)
체위성 현훈	0 (0.00)	2 (1.75)

Table 3: AEs (TEAEs) reported in ≥1% of participants in the clinical study of this product (SP-HA-003).

Group	This product group (N = 107) n (%)	Synovian group (N = 114) n (%)
Arthralgia	11 (10.28)	2 (1.75)
COVID-19	10 (9.35)	6 (5.26)
Conjunctivitis allergic	4 (3.74)	0 (0.00)
Urinary tract infection	2 (1.87)	2 (1.75)
ALT increased	3 (2.80)	1 (0.88)
Back pain	3 (2.80)	1 (0.88)
AST increased	2 (1.87)	1 (0.88)
Hyperglycaemia	2 (1.87)	1 (0.88)
Pain in extremity	2 (1.87)	0 (0.00)
Cystitis	2 (1.87)	0 (0.00)
Dyslipidaemia	2 (1.87)	0 (0.00)
Hypertension	2 (1.87)	1 (0.88)
Vertigo positional	0 (0.00)	2 (1.75)
Nasopharyngitis	0 (0.00)	2 (1.75)
Thyroid mass	0 (0.00)	2 (1.75)

Original Text [In Korean]	Translated Text [In English]
사용상의 주의사항	Precautions for Use

비인두염	0 (0.00)	2 (1.75)
갑상선 종괴	0 (0.00)	2 (1.75)

이 약을 관절강 내 1 차 투여 후 12 주 동안 또는 24 주차 재투여 받은 후 같은 기간 보고된 이상반응(TEAEs) 발현빈도는 이 약 1 차 투여 후 12 주 동안 21.50% (23/107 명), 이 약 재투여 후 12 주 동안 15.56% (14/90 명)이었으며, 이 약의 1 차 투여 또는 재투여 후 1% 이상의 발현빈도로 보고된 이상반응은 아래 표 4 와 같다.

표 4. 이 약의 임상시험(SP-HA-003)에서 1 차 투여 또는 재투여 후 12 주 동안 보고된 1 % 이상의 이상반응 (TEAEs)

구분	이 약 1 차 투여 후 (107 명) n (%)	이 약 재투여 후 (90 명) n (%)
관절통	7 (6.54)	2 (2.22)
COVID-19	3 (2.80)	1 (1.11)
알레르기 결막염	2 (1.87)	3 (3.33)
요로 감염	2 (1.87)	0 (0.00)
알라닌 아미노전이효소 증가	2 (1.87)	0 (0.00)
등허리 통증	2 (1.87)	0 (0.00)
아스파르트산 아미노전이효소 증가	2 (1.87)	0 (0.00)
혈당 증가	2 (1.87)	0 (0.00)
사지 통증	1 (0.93)	2 (2.22)
방광염	1 (0.93)	1 (1.11)
이상 지질 혈증	1 (0.93)	1 (1.11)
고혈압	1 (0.93)	0 (0.00)
체위성 현훈	0 (0.00)	1 (1.11)
비인두염	0 (0.00)	1 (1.11)
갑상선 종괴	0 (0.00)	1 (1.11)

이 약 투여군에서 중대한 이상반응은 3.74% (4/107 명)가 보고되었으며 폐동정맥루, 방광 신생물, 일과성 허혈 발작 및 과다 각화증이 각 1 명(0.93%)에서 1 건씩 보고되었다.

The incidence of AEs (TEAEs) reported within 12 weeks after the initial intra-articular administration of this product or within 12 weeks following the repeat injection at Week 24 was 21.50% (23/107) after the initial injection and 15.56% (14/90) after the repeat injection, and AEs reported with an incidence of ≥1% after either the initial or repeat administration are summarised in Table 4.

Table 4: AEs (TEAEs) with an incidence of ≥1% reported within 12 weeks after either the initial or repeat administration in the clinical study of this product (SP-HA-003)].

Group	Initial dosing (N = 107) n (%)	Re-dosing (N = 90) n (%)
Arthralgia	7 (6.54)	2 (2.22)
COVID-19	3 (2.80)	1 (1.11)
Conjunctivitis allergic	2 (1.87)	3 (3.33)
Urinary tract infection	2 (1.87)	0 (0.00)
ALT increased	2 (1.87)	0 (0.00)
Back pain	2 (1.87)	0 (0.00)
AST increased	2 (1.87)	0 (0.00)
Hyperglycaemia	2 (1.87)	0 (0.00)
Pain in extremity	1 (0.93)	2 (2.22)
Cystitis	1 (0.93)	1 (1.11)
Dyslipidaemia	1 (0.93)	1 (1.11)
Hypertension	1 (0.93)	0 (0.00)
Vertigo positional	0 (0.00)	1 (1.11)
Nasopharyngitis	0 (0.00)	1 (1.11)
Thyroid mass	0 (0.00)	1 (1.11)

In the group treated with this product, serious adverse events were reported in 3.74% (4/107) of patients, with one case (0.93%) each of pulmonary arteriovenous fistula, bladder neoplasm, transient ischaemic attack, and hyperkeratosis.

Original Text [In Korean]	Translated Text [In English]
사용상의 주의사항	**Precautions for Use**
4. 일반적 주의<br>(1) 변형성슬관절증으로 관절에 염증이 심한 경우는 이 약의 투여에 의해 국소염증 증상의 악화를 초래할 수가 있으므로 염증증상을 제거한 후 이 약을 투여하는 것이 바람직하다.<br>(2) 이 약의 투여로 매우 흔하게 국소통증, 홍반 및 종창이 나타나므로, 이 약을 관절강에 투여한 후 48 시간 동안은 격렬한 운동이나 관절에 무리가 가는 행동은 피하도록 하고, 국소안정을 지시하는 등의 조치를 한다.<br>(3) 이 약은 관절강 외에 누출되면 통증을 일으킬 우려가 있으므로 관절강 내에 확실하게 투여한다.<br>(4) 이 약은 숙련된 의사가 투여해야 한다.	4. General Precautions<br>(1) In cases of severe joint inflammation due to degenerative osteoarthritis, administration may exacerbate local symptoms. It is advisable to control inflammation prior to administration.<br>(2) Avoid strenuous activity or excessive joint use for 48 h post-injection. Patients should rest the joint as appropriate.<br>(3) Ensure accurate intra-articular administration to prevent pain due to extra-articular leakage.<br>(4) Administration should be performed by a trained healthcare professional.
5. 상호작용<br>이 약과 다른 관절 내 투여제와의 상호작용에 대한 안전성과 유효성은 입증되어 있지 않으므로 병용 투여하지 않는다.	5. Drug Interactions<br>The safety and efficacy of this product when used in combination with other intra-articular injectable products have not been established; therefore, concomitant use is not recommended.
6. 임부 및 수유부에 대한 투여<br>임부 및 수유부에 대한 이 약의 안전성 및 유효성은 확립되지 않았으므로, 이 약의 투여는 권장되지 않는다.	6. Use in Specific Populations—Pregnant or Lactating Women<br>Use is not recommended due to a lack of safety and efficacy data.
7. 소아에 대한 투여<br>소아에 대한 이 약의 안전성 및 유효성은 확립되지 않았으므로, 이 약의 투여는 권장되지 않는다.	7. Use in Specific Populations—Paediatric Patients<br>Use is not recommended as safety and efficacy have not been established.
8. 고령자에 대한 투여<br>일반적으로 고령자는 생리기능이 저하되어 있으므로 이 약 투여 시 주의한다.	8. Use in Specific Populations—Elderly<br>Caution is advised due to potentially reduced physiological function.
9. 적용상의 주의<br>(1) 이 약은 슬(무릎)관절강 내로 투여하므로 엄격한 무균조작 하에 실시한다.<br>(2) 관절액의 저류가 있을 때에는 필요에 따라 천자로 삼출물을 제거한 뒤 약물을 투여한다.<br>(3) 혈관 내, 관절 외 혹은 윤활조직에는 투여하지 않는다.<br>(4) 이 약은 내부에 동봉된 주사침을 사용하여 투여하는 것을 권장한다.<br>(5) 이 약은 살균소독제인 벤잘코늄염화물 등의 제 4 급 암모늄염 및 클로르헥시딘에 의해 침전을 일으키는 수가 있으므로 주의한다.	9. Administration Precautions<br>(1) This product is administered into the knee joint cavity and must be injected under strict aseptic conditions.<br>(2) If joint effusion is present, aspirate the exudate by arthrocentesis if necessary, before administering the product.<br>(3) Do not inject intravascularly, extra-articularly, or into synovial tissues.<br>(4) It is recommended that the enclosed injection needle be used for administration.<br>(5) Caution should be exercised, as this product may precipitate in the presence of quaternary ammonium

Original Text [In Korean]	Translated Text [In English]
**사용상의 주의사항**	**Precautions for Use**
(6) 이 약은 1 회용으로 사용하며, 재멸균 및 재사용할 수 없다. 만약 포장이나 주사기가 열려 있거나 손상이 확인 된 경우 사용해서는 안된다.<br>(7) 주사 부위는 투여 전 알코올이나 다른 적절한 소독 용액으로 소독되어야 한다.<br>(8) 투여 후 시린지, 바늘, 사용하지 않고 남은 물질은 폐기한다.<br>(9) 이 약을 양쪽 무릎에 투여하는 경우, 각각의 부위별로 주사침을 별도로 사용한다.<br>(10) 증상의 개선이 없는 경우 재투여하지 않는다.	compounds such as benzalkonium chloride or disinfectants containing chlorhexidine.<br>(6) This product is intended for single use only and must not be resterilised or reused. Do not use if the packaging or syringe is opened or damaged.<br>(7) The injection site must be disinfected with alcohol or another appropriate antiseptic solution prior to administration.<br>(8) After administration, the syringe, needle, and any unused product must be properly discarded.<br>(9) If administering to both knees, use a separate needle for each site.<br>(10) Do not re-administer if there is no symptom improvement.
10. 보관 및 취급상의 주의사항<br>(1) 어린이의 손이 닿지 않는 곳에 보관한다.<br>(2) 의약품을 원래 용기에서 꺼내어 다른 용기에 보관하는 것은 의약품 오용에 의한 사고 발생이나 의약품 품질 저하의 원인이 될 수 있으므로 원래의 용기에 보관한다.	10. Storage and Handling<br>(1) Keep out of reach of children.<br>(2) The product should be stored in its original container, as transferring it to another container may lead to medication errors or a deterioration of product quality.
11. 전문가를 위한 정보<br>(1) 약리작용<br>이 약은 외과적으로 유발된 토끼 골관절염 모델에서 관절강 내에 투여 시 슬관절의 두께 감소, 슬관절 신장 각도 증가 억제, 연골 손상 면적의 감소, Mankin score 증가 억제 및 대퇴골 및 경골 부위의 연골 두께 및 연골 세포 감소 억제 작용 및 염증성 사이토카인 IL-1β 및 TNF-α 의 활성 억제가 나타났다.<br><br>(2) 약동학적 정보<br>① 흡수: 이 약은 ^{14}C 방사성 동위원소 표지하여 수컷 랫드의 관절강 내 히알루론산나트륨으로서 2 mg/body 용량으로 투여 시 최대 혈장 농도(C_{max})는 투여 후 3 시간째 도달하였다. 혈장 내의 방사능은 투여 후 총 70 일 중 8 시간 시점까지 검출되었고, 24 시간 이후에는 검출 한계 미만이었다.<br>② 분포: 이 약은 수컷 랫드의 관절강 내 2 mg/body 용량으로 투여 시, 투여 후 56 일에서 투여부위인	11. Information for Healthcare Professionals<br>(1) Pharmacodynamics<br>This product demonstrated the following effects when administered intra-articularly in a rabbit model of surgically induced knee osteoarthritis (OA): an inhibition of joint space narrowing and increase in knee extension angle; reduction in cartilage damage area; a suppression of Mankin score increase; an inhibition of the loss of cartilage thickness and chondrocyte density in the femoral and tibial regions; and a suppression of pro-inflammatory cytokine activity, specifically IL-1β and TNF-α.<br><br>(2) Pharmacokinetics<br>① Absorption: Following an intra-articular administration of ^{14}C-labeled sodium hyaluronate at a dose of 2 mg/body in male rats, the maximum plasma concentration (C_{max}) was reached at 3 h post-dose. Radioactivity in plasma was detectable up to 8 h after administration during the 70-day observation period, but fell below the limit of detection after 24 h.<br>② Distribution: After intra-articular injection at 2 mg/body in male rats, 64.57% of the administered dose remained

**Original Text [In Korean]**	**Translated Text [In English]**
사용상의 주의사항	**Precautions for Use**
관절강에 투여량의 64.57%가 잔존하고, 그 외 간, 비장, 신장 순으로 분포되었으며, 대뇌, 갑상선, 심장, 골격근, 고환, 위에서는 투여 후 70 일까지 검출되지 않았다. 40 mg/body 용량으로 피하 또는 복강 내 투여 시 전신에 광범위하게 분포하고 시간이 지남에 따라 조직들로부터 느리게 소실되는 경향을 보였다.	at the joint site at 56 days post-dose. Distribution to the liver, spleen, and kidneys was observed, whereas no radioactivity was detected in the brain, thyroid, heart, skeletal muscle, testes, or stomach up to 70 days post-dose. When administered subcutaneously or intraperitoneally at 40 mg/body, the compound was widely distributed systemically and exhibited slow tissue clearance over time.
③ 대사: 이 약은 수컷 랫드의 복강 내 투여 후 간의 히알루로니다제에 의해 분자량 2 만에서 5 만의 대사체로 대사되며, 혈장에서 분자량 2 만에서 5 만 또는 분자량 6 천 이하의 두 가지 대사체 분획이 관찰되었다. 6 일 이후에는 주 배설경로인 소변을 통해 분자량 6 천 이하의 형태로 배설됨을 확인하였다.	③ Metabolism: Following intraperitoneal administration in male rats, the compound was metabolized in the liver by hyaluronidase into fragments with molecular weights ranging from 20,000 to 50,000. Two metabolite fractions with molecular weights of 20,000–50,000 and <6000 were detected in plasma. From day 6 onward, the compound was confirmed to be excreted in urine primarily as metabolites with molecular weights below 6000.
④ 배설: 이 약은 수컷 랫드의 관절강 내 2 mg/body 용량으로 투여 시 투여 후 70 일까지 투여부위에서 투여된 용량의 67.5%가 잔존하고, 소변과 대변으로 각각 24.9%와 2.5%가 배설됨이 관찰되었다. 40 mg/body 용량으로 피하 투여 시 투여 후 70 일까지 소변과 대변으로 23.1%와 2.4%가 배설되었고, 복강 내 투여 시 소변과 대변으로 16.4%와 1.6%가 배설되었다.	④ Excretion: Following an intra-articular administration of 2 mg/body in male rats, 67.5% of the dose remained at the injection site up to 70 days, with 24.9% and 2.5% excreted in urine and faeces, respectively. After subcutaneous administration at 40 mg/body, 23.1% and 2.4% were excreted in urine and faeces, respectively, while intraperitoneal administration resulted in 16.4% and 1.6% excretion via urine and faeces, respectively.
(3) 임상시험 정보	(3) Clinical Study Information
① 이중눈가림, 위약대조 임상 1/2 상 시험(SP-HA-001)<br>무릎(슬)관절의 골관절염 환자를 대상으로 이 약 3 mL (8 명) 또는 5 mL (8 명)을 1 회 투여 시 안전성 및 유효성을 동일한 투여 액량의 위약대조(코호트 당 2 명)와 비교 평가하기 위해, 다기관, 무작위배정, 위약대조, 이중눈가림, 2-코호트 시험이 실시되었다.<br>이 약 투여 후 12 주차 통증 점수[weight bearing pain (WBP)-100 mm Visual Analogue Scale (VAS): 체중부하 시 통증 평가] 변화량은 이 약 3 mL 투여군에서 기저치 대비 평균(표준편차) 17.71 (9.64) mm, 이 약 5 mL 투여군에서 33.14 (19.51) mm 감소하였다.	① Double-Blind, Placebo-Controlled Phase 1/2 Study (SP-HA-001)<br>A multicentre, randomised, double-blind, placebo-controlled, two-cohort trial was conducted in patients with knee OA to evaluate the safety and efficacy of a single intra-articular injection of 3 mL (N = 8) or 5 mL (N = 8) of the investigational product, which was administered and compared with an equal volume of placebo (N = 2 per cohort).<br>At Week 12, the mean (SD) change in pain score [weight-bearing pain (WBP) on a 100 mm visual analogue scale (VAS)] from baseline was -17.71 (9.64) mm in the 3 mL group and -33.14 (19.51) mm in the 5 mL group.
② 이중눈가림, 활성대조 임상 3 상 시험(SP-HA-003)<br>무릎(슬)관절의 골관절염 환자를 대상으로 이 약 및 활성대조약(시노비안주)를 1 차 투여 및 24 주차	② Double-Blind, Active-Controlled Phase 3 Study (SP-HA-003)<br>A multicentre, randomised, double-blind, active-

Original Text [In Korean]	Translated Text [In English]
사용상의 주의사항	**Precautions for Use**
재투여 시 두 군간 안전성 및 유효성을 비교 평가하기 위해, 다기관, 무작위배정, 활성대조, 이중눈가림 시험이 실시되었다. 이 약 및 활성대조약의 1 차 투여 후 12 주차 통증 점수(WBP-100 mm VAS) 변화량을 비교(PP 군)한 결과, 기저치 대비 이 약 투여군(83 명)에서 평균(표준편차) 23.71 (18.11) mm, 활성대조약 투여군(95 명)에서 25.99 (17.39) mm 감소하였으며 두 군간 비열등함을 통계적으로 입증하였다.	controlled study was conducted to compare the safety and efficacy of this product with Synovian injection. Participants received a single intra-articular injection at baseline and a repeated injection at Week 24.<br>In the per-protocol (PP) population, the mean (SD) change in WBP-VAS at Week 12 from baseline was −23.71 mm (N = 83) in the investigational product group and −25.99 mm (N = 95) in the comparator group, demonstrating statistical non-inferiority.
1 차 투여 및 24 주차 재투여 후 36 주차 통증점수 변화량을 비교(PP 군)한 결과, 기저치 대비 이 약 투여군(67 명)에서 32.22 (17.79) mm, 활성대조약 투여군(91 명)에서 29.92 (20.89) mm 감소하였다.<br>임상연구를 통해 밝혀진 기간 이외의 장기간 사용에 대한 안전성·유효성은 확인되지 않았다.	At Week 36 (post-initial and post-repeated injection), the mean (SD) change was −32.22 mm (N = 67) in the investigational product group and −29.92 mm (N = 91) in the comparator group.<br>The safety and efficacy of long-term use beyond the clinical trial periods have not been established.
4) 독성시험	4) Toxicity Studies
① 반복투여 독성시험	① Repeat-Dose Toxicity Studies
랫드를 대상으로 이 약 0.040, 0.080, 0.120 mL/head/day 용량으로 4 주 간격 8 회 반복 관절강 내 투여하여 관찰한 결과, 모든 용량에서 부검 시 시험물질에 의한 육안적 이상소견은 관찰되지 않았으며 조직병리학적 검사 결과 시험물질에 의한 변화는 관찰되지 않아, 본 시험조건에서 이 약의 무독성량(No Observed Adverse Effect Level, NOAEL)은 암수 모두 0.120 mL/head/day 로 판단하였다.	In rats, this product was administered intra-articularly at doses of 0.040, 0.080, and 0.120 mL/head/day every 4 weeks for a total of eight doses. No macroscopic abnormalities attributable to the test article were observed at necropsy at any dose, and no test article-related changes were observed in histopathological examinations. Therefore, under the conditions of this study, the no-observed-adverse-effect level (NOAEL) was determined to be 0.120 mL/head/day for both sexes.
비글견을 대상으로 이 약 0.15, 0.35, 0.7 mL/kg/day (히알루론산나트륨 기준 3, 7, 14 mg/kg/day) 용량으 로 40 주간 11 회 반복 관절강 내 투여한 결과, 모든 용량에서 독성학적으로 의미 있는 변화는 관찰되지 않아, 본 시험조건에서 이 약의 무독성량(NOAEL)은 암수 모두 14 mg/kg/day 로 판단하였다.	In beagle dogs, the product was administered intra-articularly at doses of 0.15, 0.35, and 0.7 mL/kg/day (corresponding to 3, 7, and 14 mg/kg/day of sodium hyaluronate) for a total of 11 injections over 40 weeks. No toxicologically significant changes were observed at any dose. Thus, the NOAEL was determined to be 14 mg/kg/day for both sexes under the conditions of this study.
② 유전독성시험	② Genotoxicity Studies
박테리아를 이용한 복귀돌연변이시험, CHL 세포주를 이용한 염색체이상시험, ICR 마우스 소핵시험의 유전독성 시험에서 이 약은 변이원성 및 염색체이상을 유발하지 않았다.	This product did not induce mutagenicity or chromosomal aberrations in any of the following genotoxicity tests: the bacterial reverse mutation test, the in vitro chromosomal aberration test using CHL cells, and the in vivo micronucleus test in ICR mice.

Original Text [In Korean]	Translated Text [In English]
사용상의 주의사항	**Precautions for Use**
③ 발암성시험<br>CB6F1-Tg rasH2 유전자조작 마우스를 대상으로 이 약 15, 30, 60 mL/kg (헥사메틸렌디아민으로서 9.63, 19.26, 38.52 mg/kg) 용량으로 13 주 간격 2 회 반복 복강 내 투여하여 26 주간 관찰한 결과, 암수 모두에서 최고 용량까지 발암성이 관찰되지 않았으며, 본 시험조건에서 이 약의 무독성량(NOAEL)은 헥사메틸렌디아민 기준 암수 모두 38.52 mg/kg 으로 판단하였다. 암수 고용량에서의 노출은 이 약의 임상용량에서의 사람 노출보다 약 50 배(투여량 기준) 더 높았다. 이 약의 가교제인 헥사메틸렌디아민 용액을 비교물질로 1.4 mg/head 용량으로 13 주 간격 2 회 관절강 내 주사로 투여하여 26 주간 관찰한 결과, 투여와 관련된 유의한 종양성 변화 또는 독성 변화가 관찰되지 않았다. 이 시험에서의 헥사메틸렌디아민 노출은 이 약의 임상용량에서의 사람 노출보다 약 60 배(투여량 기준) 더 높았다.†	③ Carcinogenicity Studies<br>In CB6F1-Tg rasH2 transgenic mice, the product was administered intraperitoneally at doses of 15, 30, and 60 mL/kg (equivalent to 9.63, 19.26, and 38.52 mg/kg of hexamethylenediamine) twice at 13-week intervals, and animals were observed for 26 weeks. No carcinogenicity was observed in either sex at any dose level, and the NOAEL under the study conditions was determined to be 38.52 mg/kg (based on hexamethylenediamine) for both sexes. Exposure at the highest dose corresponded to approximately 50 times the human exposure based on clinical dosing. In a separate study using hexamethylenediamine solution as a comparator, intra-articular injections were given to rats at 1.4 mg/head twice at 13-week intervals and observed for 26 weeks. No treatment-related tumorigenic or toxicological changes were observed. The exposure level in this study was approximately 60 times the human exposure based on clinical dosing. †
④ 항원성시험<br>Hartley 계 기니픽을 이용한 아나필락시스 쇼크(ASA) 반응시험 및 동종 수동 피부 아나필락시스(PCA) 반응시험에서 이 약은 항원성을 나타내지 않았다.	④ Antigenicity Studies<br>In antigenicity studies using Hartley guinea pigs, this product did not induce antigenic responses in either the active systemic anaphylaxis (ASA) test or the passive cutaneous anaphylaxis (PCA) test.
†동물 반복투여 간격(1 회/4 주)과 임상 예상 용량·용법의 차이를 고려하여 동일 기간 동물 용량(6 개월 6 회 투여노출량)과 임상 용량(6 개월 1 회 투여 노출량)을 비교하여 보정한 안전역: 투여 후 100%잔존을 가정할 경우 6 배수인 27.1, 수컷 랫드에서 의약동학데이터에 기반하여 투여 후 1680 시간에서 투여 부위인 관절강에서 투여량의 67.5%잔존과 지수소실(exponential decay)을 가정할 때 다음의 공식들에 의해 k = 0.000234 h^{-1}, 소실반감기($t_{1/2}$)는 2962.8 시간(123.5 일)이고, 6 회 투여 시 잔존량은 4.2 배수인 19.0 으로 산출됨	†Safety margin adjusted for differences in dosing interval and regimen between animal and clinical studies, considering the difference in dosing intervals between animals (once every 4 weeks) and the anticipated clinical regimen; the safety margin was adjusted by comparing the cumulative exposure over the same period (six doses over 6 months in animals vs. one dose over 6 months in humans): Assuming 100% retention after each dose, the resulting estimated safety margin is 27.1, which is 6-fold. Assuming the same pharmacokinetic profile observed in male rats—67.5% of the dose remains at the administration site (joint cavity) at 1680 h post-dose with exponential decay—the following calculations apply: elimination rate constant (k) = 0.000234 h^{-1}, elimination half-life ($t_{1/2}$) = 2962.8 h (123.5 days). Under these assumptions, residual amount after six doses yields an estimated safety margin of 19.0, which is 4.2-fold.

References

1. Ha, C.W.; Park, Y.B.; Choi, C.H.; Kyung, H.S.; Lee, J.H.; Yoo, J.D.; Yoo, J.H.; Choi, C.H.; Kim, C.W.; Kim, H.C.; et al. Efficacy and safety of single injection of cross-linked sodium hyaluronate vs. three injections of high molecular weight sodium hyaluronate for osteoarthritis of the knee: A double-blind, randomized, multi-center, non-inferiority study. *BMC Musculoskelet. Disord.* **2017**, *18*, 223. https://doi.org/10.1186/s12891-017-1591-4. PMID: 28549436.
2. Product Overview and Prescribing Information for Hyalflex® (Sodium Hyaluronate Gel Cross-linked Hexamethylenediamine). Available online: https://nedrug.mfds.go.kr/pbp/CCBBB01/getItemDetailCache?cacheSeq=202401722 (accessed on 3 June 2025). (In Korean)
